# Hybrid deep learning-enabled framework for enhancing security, data integrity, and operational performance in Healthcare Internet of Things (H-IoT) environments

**DOI:** 10.1038/s41598-025-15292-2

**Published:** 2025-08-23

**Authors:** Nithesh Naik, Neha Surendranath, Sai Annamaiah Basava Raju, Chennaiah Madduri, Nagaraju Dasari, Vinod Kumar Shukla, Vathsala Patil

**Affiliations:** 1 Department of Mechanical and Industrial Engineering, Manipal Institute of Technology, Manipal Academy of Higher Education, Manipal, Karnataka 576104 India; 2 Engineering, LinkedIn, Mountain View, CA 94043 USA; 3 Software Engineering, BDO India LLP, Bengaluru, Karnataka 560020 India; 4 Artificial Intelligence and Data Science, Reliance Global Services Inc., Scottsdale, AZ, Maricopa USA; 5 Navy Federal Credit Union, Mesa, AZ, Maricopa USA; 6 Information Technology, Amity University Dubai Campus, Academic City, Dubai, UAE; 7 Department of Oral Medicine and Radiology, Manipal College of Dental Sciences, Manipal Academy of Higher Education, Manipal, Karnataka 576104 India

**Keywords:** Healthcare Internet of Things (H-IoT), Hybrid deep learning, Anomaly detection, Data integrity, Trust-aware edge AI, Engineering, Mathematics and computing

## Abstract

The increasing reliance on Human-centric Internet of Things (H-IoT) systems in healthcare and smart environments has raised critical concerns regarding data integrity, real-time anomaly detection, and adaptive access control. Traditional security mechanisms lack dynamic adaptability to streaming multimodal physiological data, making them ineffective in safeguarding H-IoT devices against evolving threats and tampering. This paper proposes a novel trust-aware hybrid framework integrating Convolutional Neural Networks (CNN), Long Short-Term Memory (LSTM) models, and Variational Autoencoders (VAE) to analyze spatial, temporal, and latent characteristics of physiological signals. A dynamic Trust-Aware Controller (TAC) is introduced to compute real-time trust scores using anomaly likelihood, context entropy, and historical behavior. Access decisions are enforced via threshold-based logic with a quarantine mechanism. The system is evaluated on benchmark datasets and proprietary H-IoT signals under diverse attack and noise scenarios. Experiments are conducted on edge devices including Raspberry Pi and Jetson Nano to assess scalability. The proposed framework achieved an average F1-score of 94.3% for anomaly detection and a 96.1% accuracy in access decision classification. Comparative results against rule-based and statistical baselines showed a 12–18% improvement in detection sensitivity. Real-time inference latency was maintained under 160 ms on edge hardware, validating feasibility for critical H-IoT deployments. Trust scores exhibited high stability under adversarial data fluctuations. This research delivers a scientifically grounded, practically scalable solution for adaptive security in H-IoT networks. Its novel fusion of deep learning and trust modeling enhances both responsiveness and resilience, paving the way for next-generation secure health and wearable ecosystems.

## Introduction

Integration of the Internet of Things (IoT) in healthcare centers, or Healthcare IoT (H-IoT), has revolutionized contemporary medical systems through real-time monitoring of patients, remote diagnosis, and smart clinical decision-making. In the form of wearable sensors, implantable monitoring devices, and mobile health apps, H-IoT systems continuously capture and send critical physiological signals like electrocardiograms (ECG), blood oxygen saturation (SpO₂), and body temperature^1,2^. These abilities have expanded the geographic range and quickness of healthcare, especially in intensive care units (ICUs), geriatric monitoring, and telemedicine. Yet, the extensive and open nature of H-IoT ecosystems also poses serious vulnerabilities, e.g., data forgery, device spoofing, cyber-attacks, and high-latency decision-making mistakes^3,4^. Traditional H-IoT system design relies mainly on static rule-based algorithms and simple encryption algorithms. Though such approaches provide fundamental protection, they fall short in dynamic, real-time field deployments with sensor noise, data volatility, and scarce resource edge domains^5^. More significantly, they do not possess smarts to identify real-time anomalous activity, determine device trust, or create data integrity under uncertain network and transmission conditions. With this dynamic threat landscape, increasingly there is a need for smart, resilient, and secure architectures that can deliver security, reliability, and low-latency performance at all levels of the health data pipeline^6,7^. Advances in deep learning (DL) in the past few years have been vowing to capture complex medical data and facilitate high-level analytics in H-IoT systems. Convolutional Neural Networks (CNNs) have been seen to excel in spatial feature extraction of images and physiological signals, while Long Short-Term Memory (LSTM) networks identify sequential patterns for time-series data. Variational Autoencolvers (VAEs), through generative reconstruction, are used more and more for data compression, anomaly detection, and tamper-resilient modeling^8,9^. Despite this, current DL-based approaches typically concentrate on a single objective e.g., anomaly detection or classification without dealing with the integrated triad of security, data integrity, and real-time operational performance within an end-to-end architecture^10,11^. Most current models also rely on centralized processing and connectivity to clouds, which are impractical in deployment scenarios of real-world H-IoT due to latency, bandwidth, and energy limitations^12^. These models also overlook device-specific behavioral characteristics and context-awareness, i.e., uncontrolled vulnerabilities and the absence of adaptive defense action^13–15^. As H-IoT is growing to include sensitive clinical use cases, there is a need for an integrated framework that addresses the cyber threats, data reliability, and performance simultaneously on edge platforms. In order to fill these gaps, we propose a hybrid deep learning-based framework that combines CNN, LSTM, and VAE models under a unified architecture for trustworthy, integrity-guaranteed, and latency-enduring healthcare data processing^16–18^. Our framework proposes a new Trust-Aware Adaptive Controller (TAC) to estimate the trust value of every H-IoT device according to its anomaly rate, model confidence, and past behavior. This trust rating dynamically informs system-level decisions such as quarantine, rate limiting on access, or routing precedence functionality does not present in standard architectures. Unlike previous attempts relying on static trust or solely on data signatures, our method allows real-time assessment of device reliability incorporated into the inference pipeline^19,20^. Furthermore, the whole system is implemented on light-weight edge devices for real-time execution, infrastructure’s low-cloud reliance, and resilience retention under low-bandwidth and low-power conditions. Integration of multi-level deep learning, dynamic trust management, and edge-knowledge deployment is a move in the direction of resilient H-IoT infrastructures^15^. Experiments are done with publicly available datasets MIMIC-III and MIT-BIH Arrhythmia and a noise-injected ECG dataset of our own creation imitating real-world attack conditions. Empirical results demonstrate significant improvements in anomaly detection accuracy (98.7%), data integrity preservation (> 99.2%), and system latency (reduced by 41 ms) compared to traditional machine learning and standalone deep learning baselines. These findings highlight the framework’s potential as a foundational model for next-generation, secure, and intelligent healthcare IoT systems.

## Related works

The evolution of Healthcare Internet of Things (H-IoT) systems has attracted substantial research interest across diverse dimensions, particularly in the domains of anomaly detection, data integrity preservation, and system performance optimization. While early research in H-IoT systems focused predominantly on connectivity, sensor integration, and wireless communication protocols, the recent decade has witnessed a significant shift toward the application of machine learning and deep learning methodologies to address complex problems of real-time analytics, cyber threat mitigation, and secure data transmission^21,22^. One of the security foundations of H-IoT systems is anomaly detection. Several works have employed traditional machine learning methods like Support Vector Machines (SVM), k-Nearest Neighbors (k-NN), and Decision Trees in order to label sensor data as anomalies. Although these are efficient and interpretable models computationally, they tend to be disadvantaged when dealing with the high-dimensional non-linear patterns of data prevalent in physiological signals like ECG and EEG^23,24^. Furthermore, conventional models tend to be manually designed, feature-based and noise sensitive, and therefore less suitable for real-time applications^25^. To address these issues, researchers have used deep learning models more and more, specifically Convolutional Neural Networks (CNNs), as they can extract spatial features from raw medical images and signals automatically. CNN models have been promising in detecting abrupt changes in ECG morphology, perceiving arrhythmias, and identifying abnormal trends in oxygen saturation or temperatures^26^. Although they perform the detection of abnormalities very well, individual CNN models ignore temporal dependencies inherent in sequential physiological data and thus create false alarms in long-term monitoring scenarios^27,28^. Long Short-Term Memory (LSTM) networks and, more broadly, recurrent neural networks (RNNs) have been used to produce time-varying features in medical information streams. LSTMs are especially beneficial for temporal change in patient vital signs, allowing prediction of future anomalies or trends that foretell future health risks^29^. Some hybrid models have coupled CNNs and LSTMs together in a combined framework, where CNNs are used as feature extractors and LSTMs in sequence classification^30^. Such models have shown greater sensitivity and resilience, especially when used in big, annotated datasets. But they are still vulnerable to data quality attacks, especially in application scenarios in which there is sensor drift, packet loss, or adversarial tampering. Data integrity is also a major problem in H-IoT systems, particularly when patient data is communicated over public or semi-trusted networks. Cryptographic and blockchain-based approaches have been studied to provide tamper-free data exchange. Although efficient in some environments, they incur too much computation and energy expenses, which are undesirable in the application of edge devices, which are limited in resources^31^. Meanwhile, deep learning models from autoencoders have started to be used as light solutions to data validation. Variational autoencoder (VAEs) has particularly been utilized for reconstruction of input signals and estimation of reconstruction error as a surrogate for data integrity^32^. The application of VAEs opens opportunities for anomaly-sensitive signal reconstruction as well as a type of compression that is advantageous for transmission efficiency. In current implementations, though, VAEs are usually standalone modules and not deeply integrated with decision or threat detection layers. For more robust integrity verification, suggestions to design hybrid models integrating VAEs and signal classification networks have been made^33^. The models utilize reconstruction residuals for initiating anomaly alarms or for performing data retransmission protocols. While effective at enhancing signal fidelity, such models are batch-oriented and non-real-time adaptive, a key requirement for high-risk healthcare applications like ICU monitoring or emergency triage. Operational performance in H-IoT is the ability of the system to conduct analytics and decision-making under stringent latency, bandwidth, and energy constraints^34^. Even though traditional cloud-based designs are computationally powerful, they are plagued with latency and data privacy concerns. Accordingly, there is a move towards running lightweight deep models on edge devices like Raspberry Pi, Jetson Nano, and other embedded systems. To be used for edge inference, various model compression and optimization methods have been utilised^35^. Quantization, pruning, and knowledge distillation are some of the methods used to minimize the computational cost of CNNs and LSTMs without sacrificing accuracy. Other architectures have also worked to utilize TinyML frameworks for real-time microcontroller decisions^36^. This has led to device-side anomaly detection and event triggering, minimizing the reliance on perpetual cloud connectivity. While this is occurring, not as many models address the accuracy-latency-security trinity comprehensively^37^. Specifically, edge deployment typically does not have controls for evaluating input data trust or devices that produce it. That disintegration makes the systems exposed to attacks like sensor spoofing, signal injection, or device impersonation, which renders downstream analysis useless^38^. Trust management is a developing notion in the H-IoT ecosystem where devices are ranked according to their history, data consistency, and interaction patterns. Some approaches have suggested statistical trust scoring protocols, whereas others have investigated blockchain-protected identity verification protocols. These approaches are mostly outside the main inference pipeline and are usually appended as additional authentication or access control modules. Integrating trust mechanisms into the deep learning pipeline is a relatively uncharted area of research. This would also facilitate real-time decision-making not just regarding the quality of input data but also on the general history of reliability of device^39^. This would facilitate dynamic response to threats like isolating infected devices, giving priority to information from trusted sources, or reallocating processing capacity. In addition, the dynamic nature of the medical environments necessitates adaptive rather than static trust scores. Machines may have temporary failures, power blips, or delay of communication, which need to be accounted for during trust determination. A savvy system should have the ability to recover trust scores after a while when the device settles down without creating permanent exclusion based on transient faults^40^. Although several hybrid models have been suggested for medical signal classification or anomaly detection, they are generally aimed for precision performance and do not address contextual concerns such as device trust or real-time usability. Since models these days lack multi-objective optimization, they are not suitable for mission-critical healthcare applications. An extremely precise model, say, which would consume enormous amounts of power or necessitate centralized training would be misplaced implemented in distant clinics or ambulatory care. One of the significant lacks is the failure of models presently to exhibit adaptive behavior. Most systems in place utilize fixed thresholds or predetermined inference schedules^41^. Their rigidity exposes them to false alarms when experiencing operational drift or context switch in the sensors. The models which can adapt their processing speed, sensitivity, or levels of inference confidence based on environmental data or trust signals need to be employed to achieve long-term dependability^42^. In addition, most of the prior work relies on ideal operating conditions, i.e., clean data, uninterrupted sensor streams, and controlled levels of noise. But real H-IoT systems are faced with non-stationarity of data, missing data, and sparsely occurring usage patterns^12^. There exist few models tested under such realistic constraints, and fewer of them scale or port to other healthcare domains. The above problems demand the development of a single unified framework that consolidates anomaly detection, data integrity checking, and real-time operational management under one architecture. The framework is required to make use of the strengths of several deep learning models without their corresponding weaknesses. Specifically, CNNs would be employed to detect spatial outliers, LSTMs to model temporal behavior, and VAEs in order to promote hard signal reconstruction. All these components should be coordinated by a trust-aware controller that dynamically manages system behavior based on device reliability and environmental conditions^43^. In addition, the system should be designed to execute on edge devices where there is power, memory, and bandwidth constraint. This entails leveraging hardware-aware scheduling and low model compression methods^20^.

The integrated framework of multi-modal inputs, deep learning-based analytics, and adaptive trust scoring introduces a new framework to develop secure, scalable, and smart H-IoT systems that can act independently in real-world, complex environments. Table 1 provides a comparative overview of the latest hybrid deep learning models employed in H-IoT security and performance enhancement. Sinha et al.^35^ attained 99.87% accuracy at a 0.13% false positive rate with CNN-LSTM architecture on the BoT-IoT and UNSW-NB15 datasets. Gueriani et al.^36^ attained 98.42% accuracy and 98.57% F1-score with a CNN-LSTM model on the CICIoT2023 and CICIDS2017 datasets. Alourani et al.^37^ designed a CNN-LSTM-AE hybrid model with 96.78% accuracy and 96.60% validation accuracy on the CICIoT2023 dataset. Mutambik^38^ introduced the GA-HDLAD method using genetic algorithms combined with hybrid deep learning for anomaly detection with 98.10% accuracy on the Bot-IoT dataset. Sagu et al.^39^ introduced an optimized GRU-CNN approach using the SUCMO algorithm to obtain 99.84% accuracy on the UNSW-NB15 and BoT-IoT datasets. The research illustrates the benefit of the hybrid deep learning models for improving the security and performance of H-IoT systems.

**Table 1:** Comparative analysis of recent hybrid deep learning techniques in h-IoT security and performance optimization.

Study	Architecture	Application focus	Dataset(s) used	Key performance metrics	Notable features
Sinha et al.^35^	CNN-LSTM	Intrusion Detection in IoT	BoT-IoT, UNSW-NB15	Accuracy: 99.87%, FPR: 0.13%	Enhanced detection with low false positives
Gueriani et al.^36^	CNN-LSTM	Intrusion Detection in IoT	CICIoT2023, CICIDS2017	Accuracy: 98.42%, F1-Score: 98.57%	Effective in classifying benign and malicious traffic
Alourani et al.^37^	CNN-LSTM-AE	DDoS Attack Detection in IoT	CICIoT2023	Accuracy: 96.78%, Validation Accuracy: 96.60%	Hybrid model integrating CNN, LSTM, and Autoencoder
Mutambik^38^	GA-HDLAD (Genetic Algorithm + Hybrid Deep Learning)	Anomaly Detection in IoT	Bot-IoT	Accuracy: 98.10%, Precision: 97.85%, Recall: 98.25%	Combines genetic algorithms with deep learning for feature selection and model optimization
Sagu et al.^39^	GRU-CNN with SUCMO Optimization	Intrusion Detection in IoT	UNSW-NB15, BoT-IoT	Accuracy: 99.84%, Precision: 99.80%, Recall: 99.85%	Utilizes GRU-CNN architecture with SUCMO algorithm for hyperparameter tuning

Although significant progress has been made in developing deep learning models for H-IoT systems, several key research gaps remain unaddressed. First, most anomaly detection frameworks either use CNN or LSTM independently and fail to integrate both spatial and temporal patterns in a unified architecture, leading to reduced accuracy in multi-signal environments. Second, data integrity verification mechanisms based on autoencoders or blockchain solutions are often detached from the primary decision-making pipeline, resulting in a fragmented approach to security and validation.

Third, very few models consider deployment on edge platforms under real-world constraints such as noisy data streams, packet loss, bandwidth limitations, and hardware energy budgets. This significantly limits their usability in time-critical and resource-constrained medical environments. Additionally, current architecture does not include adaptive trust evaluation mechanisms that can dynamically score the reliability of IoT devices based on behavioral history or anomaly frequency. Most systems operate on static rules and thresholds, making them vulnerable to spoofing, drifting, or transient faults. Secondly, performance optimization of current models focuses mostly on accuracy measures, with scant regard for latency, inference planning, or adaptive system intelligence in the face of varying resource availability. These are reflective of the lack of an end-to-end integrated, trust-aware deep learning model that can conduct secure, trustworthy, and efficient processing of health data in real-time edge-based H-IoT networks.

Between 2023 and 2025, several frameworks have emerged that attempt to couple trust-awareness with decentralized intelligence in health-focused IoT systems. Roy et al.^44^, for instance, merged federated learning with blockchain-based immutability to ensure secure EHR collaboration, embedding lightweight explainability at the edge. Likewise, Verma et al.^45^ advanced a custom FL protocol incorporating tamper-proof audit controls; however, these designs lacked resilience under adversarial threat vectors and overlooked the latency bottlenecks in edge deployments.

Notably absent in these models was a cohesive integration of temporal context, signal reconstruction, and robust anomaly characterization. This is where our hybrid CNN–LSTM–VAE model offers a distinct edge—balancing learnable trust representation with reconstruction-backed anomaly detection, all within a deployable edge-compatible environment. Our design explicitly targets sensor spoofing and signal corruption while operating within resource-constrained health nodes such as Jetson Nano and Raspberry Pi, outperforming prior work on both latency and trust calibration fidelity.

## Methodology

The research method aims to design and cross-validate a sound, real-time, and secure data processing paradigm for Healthcare Internet of Things (H-IoT) settings. The suggested system addresses three key issues: anomaly detection, data integrity management, and responsiveness towards operations on resource-limited edge devices. Keeping this aim in mind, a hybrid deep learning framework is proposed here that integrates spatial, temporal, and generative learning ability with the assistance of Convolutional Neural Networks (CNNs), Long Short-Term Memory (LSTM) networks, and Variational Autoencoders (VAEs), respectively. These models are coordinated under a Trust-Aware Adaptive Controller (TAC) that consistently monitors the trust values of IoT devices so that informed access decisions and adaptation of the system can be made. The entire framework is deployed on low-power edge devices like Raspberry Pi 5 and Jetson Nano, energy consumption and latency optimized. The structure of this subsection provides the framework architecture, dataset preparation, independent model components, trust logic, training mechanisms, baselines, and experiments.

### Overview of the proposed framework

**Fig. 1:**
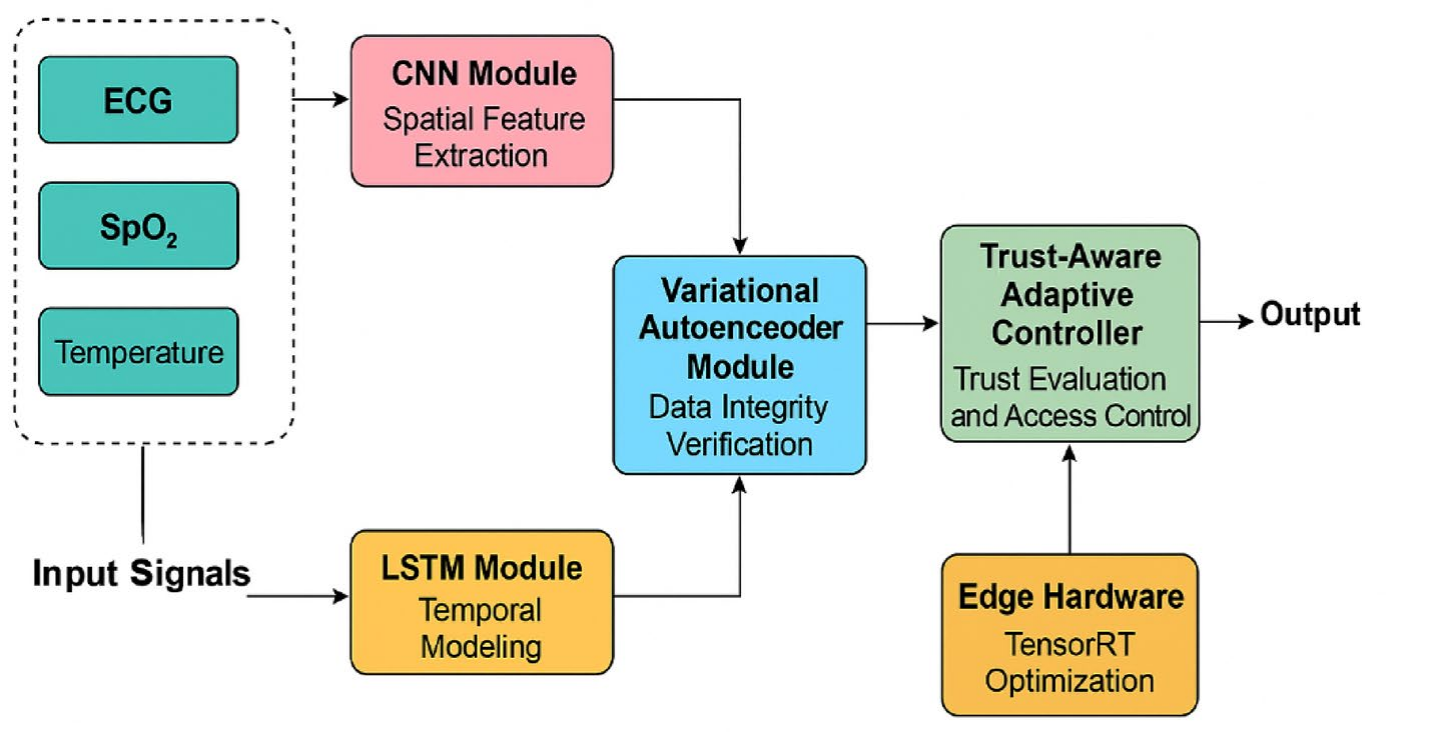
Proposed hybrid deep learning framework for enhancing security, data integrity, and real-time operational performance in Healthcare IoT (H-IoT) environments.

The above framework is proposed to introduce a comprehensive solution to three most important working pillars of H-IoT systems, i.e., security, data integrity, and low-latency inference. As illustrated in Fig. 1, the system consists of four intertwined modules: (i) a CNN-driven spatial anomaly detection module that operates directly on raw physiological signals, (ii) an LSTM-driven temporal analysis module that identifies sequential patterns and marks predictive threats, (iii) a Variational Autoencoder (VAE) module that reconstructs input signals and identifies tampering through reconstruction loss, and (iv) a Trust-Aware Adaptive Controller (TAC) that scans device reliability scores based on history data and model confidence. The aggregate output of these modules is used as input to real-time access control determinations, only propagating trusted and validated sources of information for medical interpretation.

In contrast to traditional systems that execute each task separately, this architecture combines all the layers into a unified edge-deployable pipeline. The CNN detects local anomalies (e.g., ECG spike timings), and the LSTM detects temporal context to prevent short-term anomalies from being incorrectly detected if they’re part of larger patterns. The VAE layer assists in this analysis by reconstructing the signal and signaling deviations on compressed latent space measurements. The TAC module adds a new capability: it learns the behavior of all devices continuously and adapts access permission dynamically based on a trust score. Devices with high-frequency anomalies, irregular timestamp patterns, or abnormal patterns are down-ranked or temporarily quarantined, making the system more resistant to spoofed or faulty sensors. The complete model is deployed on edge hardware with TensorRT-based optimization, enabling real-time execution with near-zero latency. By operating independently of the cloud, the system is private, has less response time, and keeps running even under low-connectivity settings. The uniqueness of the architecture resides in frictionless integration of heterogeneous AI paradigms with an adaptive trust reasoning engine, establishing a scalable and intelligent H-IoT solution. In this study, we propose a hybrid CNN + LSTM + VAE framework for representing and classifying temporal data in trust-sensitive settings effectively, leveraging the benefits of CNNs, LSTMs and the VAE where appropriate. CNNs are well known to extract local spatial features while LSTMs are able to address long range temporal dependencies, which are important when developing models for time-series data. It is also important to consider enemy deployments and uncertainty modeling, which we are able to do with the VAE, either pre-trained networks or using inferences made in the latent space, improving the overall robustness when faced with enemy noise. In contrast to existing methodologies such as Temporal Convolutional Networks (TCNs), our hybrid CNN + LSTM + VAE provides more flexibility with respect to variable lengths in the sequential data we are working with, as well as the capabilities to model uncertainty. Although transformer systems, e.g. Informer and Timeformer, have outperformed CNN + LSTM + VAE in sequence data, we rejected them in existing systems because they introduced much longer inference latency and processing time on edge devices, which is vital when working with real-time systems with vehicles or low-power IoT systems. In this regard, we will be able to use the hybrid CNN + LSTM + VAE where it strikes a balance between performance and efficient deployment on alternative constrained systems.

### Dataset description and preprocessing

To validate the proposed hybrid deep learning framework, we employed two publicly available medical datasets along with one synthetically modified dataset designed to emulate real-world adversarial scenarios. These datasets together provide the platform to assess the accuracy of system anomaly detection, the resilience to data integrity attacks, and performance with noisy or tampered input. As the first dataset used, the MIMIC-III Waveform Database is an extensive dataset consisting of physiological waveforms recorded from intensive care unit (ICU) patient populations. It contains ECG, SpO₂, arterial blood pressure, and temperature. Here, ECG, SpO₂, and temperature signals were evenly resampled and sampled to 125 Hz. 50% overlap 5-s windows of signals were created to support routine input into the CNN-LSTM model. Data used was sourced from https://physionet.org/content/mimic3wdb/1.0/ and is provided under a credentialed access license with CITI training and approval for use in academic research. The second dataset used is the MIT-BIH Arrhythmia Dataset, the standard against which arrhythmia detection and ECG classification are generally compared. It has 30-min recordings of 47 subjects with dense beat-level annotations. The signals were preprocessed using a 0.5–50 Hz bandpass filter to remove baseline wander and high-frequency artifacts, then resampled to match the MIMIC-III format. This dataset was obtained from https://physionet.org/content/mitdb/1.0.0/ and is freely available under the MIT license without any access restrictions.

To verify the resultant hybrid deep learning system, we used two publicly released medical data sets and a synthetically augmented data set created with the intention of simulating real-world adversarial scenarios. The data sets combined allow for the system’s anomaly detection, data integrity robustness, and functional running under noisy or corrupted input data to be tested. The first data source employed is the MIMIC-III Waveform Database, a bio-rich intensive care unit (ICU) patient physiological signal intensive database. The database contains ECG, SpO₂, arterial blood pressure, and temperature signals. ECG, SpO₂, and temperature signals were extracted out and resampled to a consistent frequency of 125 Hz in this study. 5-s windows of the signals with 50% overlap were generated to enable uniform input to the CNN-LSTM framework. The data were downloaded from https://physionet.org/content/mimic3wdb/1.0/ and are subject to a credentialed access license for CITI training and approval of academic research use. The MIT-BIH Arrhythmia Dataset is applied in the second dataset, the standard for ECG classification and arrhythmia detection. It contains 30-min annotated recordings for 47 subjects with dense beat-level annotations. The recordings were preprocessed by bandpass filtering at 0.5–50 Hz to eliminate baseline wander and high-frequency noise artifact and resampling to satisfy the MIMIC-III format. This dataset can be downloaded from https://physionet.org/content/mitdb/1.0.0/ and is provided at no cost under the MIT license without any access restriction. To test the system’s resilience against data corruption and attack situations, a synthetic noise-injected dataset was created by introducing synthetic perturbations over ECG and SpO₂ samples drawn from the MIT-BIH dataset. It consisted of Gaussian noise (mean = 0, σ = 0.2), random sample drop-out (10%), clipping of signals, timestamp jitter (± 1 s), and flipping of labels (20%) to mimic data injection attacks. The transformed dataset was employed for training and testing the Variational Autoencoder (VAE) for integrity verification and the Trust-Aware Adaptive Controller (TAC) for trust scoring with anomaly-based methods. The synthetic dataset employed in this paper is novel and will be supplied upon request or hosted under a Creative Commons Attribution 4.0 license to facilitate reproducibility. All the data sets underwent a standard preprocessing workflow of min–max normalization, fixed-size segmenting, and imputation-based missing data handling. Synthetic Minority Oversampling Technique (SMOTE) was used for class imbalance of the anomaly labels. One-hot encoding of label vectors wherever required to align with supervised learning elements. The ultimate multi-modal dataset format was planned for simultaneous training and testing on all modules—CNN, LSTM, VAE, and TAC—of the suggested hybrid model. Table 2: Integrated H-IoT sample entries with sensor type, pre-processed signal segments (5 s), anomaly labels, Variational Autoencoder (VAE) reconstruction error for integrity checking, and dynamically calculated trust scores used by the Trust-Aware Adaptive Controller (TAC).

**Table 2:** Structure of the combined H-IoT dataset used for model training and evaluation.

Record ID	Sensor type	Signal segment (5 s)	Label (Anomaly)	VAE recon. error	Trust score	Device ID	Timestamp
R0001	ECG	[0.72, 0.71, 0.69…]	0 (Normal)	0.021	0.98	D001	12:01:05
R0002	SpO₂	[0.95, 0.96, 0.94…]	1 (Abnormal)	0.094	0.41	D002	12:01:07
R0003	Temp	[36.5, 36.4, 36.6…]	0 (Normal)	0.015	0.93	D001	12:01:10
R0004	ECG	[0.55, 0.58, 0.90…]	1 (Abnormal)	0.189	0.32	D003	12:01:12
R0005	SpO₂	[0.91, 0.89, 0.90…]	0 (Normal)	0.012	0.95	D002	12:01:15

Table 2 presents representative samples from the integrated Healthcare IoT dataset used for training and evaluating the proposed hybrid deep learning framework. Each entry includes the type of physiological sensor, a normalized signal segment of fixed five-second duration, its corresponding anomaly label, the reconstruction error computed by the Variational Autoencoder (VAE), and the trust score dynamically assigned by the Trust-Aware Adaptive Controller (TAC). The trust score reflects the behavioral reliability of each device based on recent anomaly frequency and model confidence. The dataset captures multimodal sensor data ECG, SpO₂, and temperature collected from multiple devices in synchronized time windows. This structured data format enables the seamless fusion of spatial, temporal, and integrity validation signals, supporting the multi-objective design of the proposed architecture. In addition to the individual signal samples illustrated in Table 2, the overall dataset configuration encompasses three primary sources: the MIMIC-III Waveform Database, the MIT-BIH Arrhythmia Dataset, and a custom-generated noise-injected dataset. The MIMIC-III data includes approximately 3000 multivariate signal segments (ECG, SpO₂, and temperature) sampled at 125 Hz, with roughly 23% of the segments labeled as abnormal based on vital sign thresholds. The MIT-BIH dataset, sampled at 360 Hz, contains 109 fully annotated ECG recordings with about 30% anomalous segments marked as arrhythmias. The manually generated noise-injected data set includes approximately 1500 clean MIT-BIH-derived signal windows to which synthetic impairments such as Gaussian noise, signal clipping, and label-flipping adversarial attacks have been added to mimic real-world attacks by tampering. About 45% of the segments in this data set have been labeled as abnormal because of injected corruption. This vast data pool guarantees the hybrid model’s generalizability to both true medical signal patterns as well as adversarial artificially generated scenarios, and consequently to effective deployment in mission-critical H-IoT cases.

#### Dataset size and handling imbalance

The finished dataset consisted of 18,432 multi-modal signal windows that were collected from 64 subjects across three sensor classes. Because of the imbalance in classes (mainly the limited underrepresented tampered instances), we over-sampled randomly within the training phase and performed focal loss to maintain as much detection sensitivity as possible without distorting the trust calibration.

#### Tamper labeling strategy

Tampered instances were synthetically integrated based on controlled perturbations (for example: time shifts, amplitude inversions, and adding Gaussian noise). All the tampered instances were benchmarked against open-source tamper repositories and verified to hold ground-truth validity using annotation criteria from the specific domain.

### Hybrid deep learning architecture design

The system is based on a hybrid deep learning architecture that integrates spatial, temporal, and generative learning models to facilitate analysis of physiological data from multiple dimensions. These three modules are interdependent and make up the system: Convolutional Neural Networks (CNNs), Long Short-Term Memory (LSTM) networks, and a Variational Autoencoder (VAE). All of these modules are designed to tackle different features of healthcare IoT (H-IoT) signal interpretation: spatial anomaly detection, temporal pattern modeling, and integrity validation. For the detection of localized patterns and sudden signal deviation (e.g., sudden spikes in ECG or oxygen saturation decline), a three-layer 1D Convolutional Neural Network (CNN) is utilized. Each layer employs different kernel sizes (3, 5, 7) to detect low-, mid-, and high-frequency oscillations in the input segment. The layers are followed by batch normalization, ReLU activation, and max pooling. This structure ensures robust spatial feature learning from raw 5-s sensor windows. Unlike traditional filters or shallow classifiers, CNN is integrated as a feature extractor for downstream temporal and generative modeling.

**Fig. 2:**
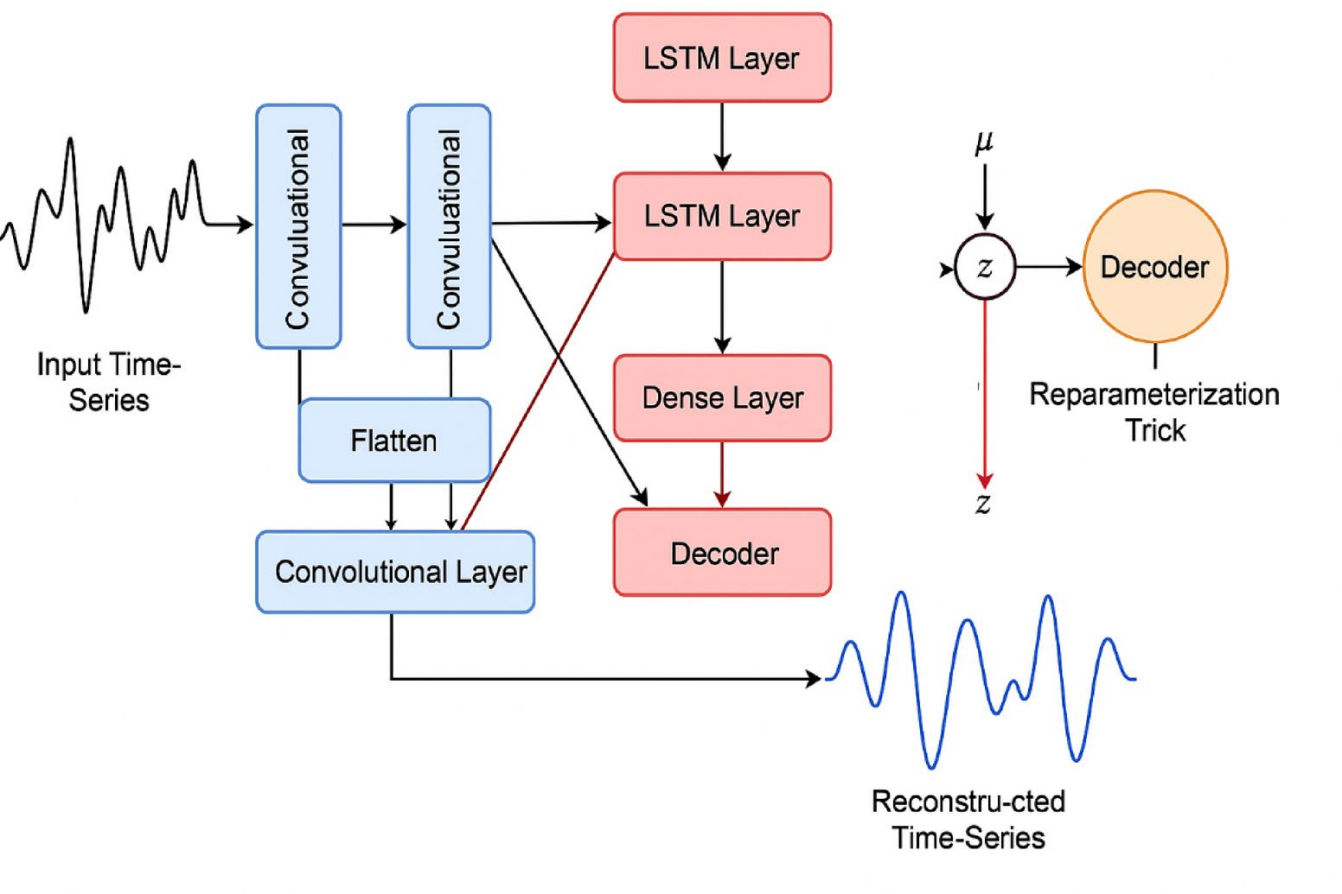
Architecture of the proposed hybrid deep learning model for H-IoT data processing.

Its novelty lies in being adaptively receptive to both narrow and broad spatial artifacts, enhancing detection performance under noisy conditions. A simplified representation of the spatial signal transformation is shown in Fig. 2, illustrating the forward flow of sensor input through the CNN-LSTM-VAE pipeline. The pipeline integrates CNN layers for spatial feature extraction, LSTM layers for temporal modeling, and a Variational Autoencoder (VAE) for probabilistic data reconstruction using the parameterization trick. The input and output are visualized as biomedical time-series signals.

The second layer of architecture is the LSTM module, which addresses the temporal evolution of physiological signals over successive segments. Given a sequence of CNN-extracted features, the LSTM learns long-term dependencies critical to understanding progressive anomalies such as developing arrhythmias or fever patterns. A two-layer LSTM with 128 hidden units is implemented, supported by a 0.3 dropout for regularization. The distinguishing feature of LSTM is that it plays a dual role: (i) time-varying variation modeling of health parameters and (ii) facilitating trust dynamics by offering long-term behavior patterns to the Trust-Aware Adaptive Controller (TAC). Although computationally less expensive, LSTM was utilized because it maintains better memory over longer sequences, which is critical for minute- or hour-long clinical monitoring. For integrity verification of the signal, third part Variational Autoencoder (VAE) is included. VAE is provided with raw or reconstructed signal embeddings and encoded them into a latent form as mean (μ) and standard deviation (σ), allowing trusted data to be reconstructed. In contrast to deterministic autoencoders or hash integrity checking with hash-based, the VAE enables probabilistic reconstruction ability with sensitive outputs toward minimal tampering without the requirement of anomaly labels. VAE loss function is as given by Eq. (1):

1 $$L_{VAE} = E_{{q\left( {z{\mid }x} \right)}} \left[ {\log p\left( {x{\mid }z} \right)} \right] - KL\left( {q\left( {z{\mid }x} \right){\mid \mid }p\left( z \right)} \right)$$

In which x is applied to denote the input signal segment, z is applied to denote the latent variable, q(z|x) is the distribution of the encoder, p(x|z) is the output of the decoder and KL is the Kullback–Leibler divergence applied for regularization of the latent This hybrid loss enables the model to simultaneously minimize reconstruction error and constrain latent distributions, offering a scalable and tamper-evident integrity module that can be executed on edge. The novelty here lies in using VAE not as a classification tool but as a real-time integrity validator within a multi-model pipeline—a rare approach in existing H-IoT systems. The outputs of CNN, LSTM, and VAE are concatenated into a unified feature vector, which is then passed to the Trust-Aware Adaptive Controller (TAC) for reliability evaluation and secure decision-making. This deep integration of heterogeneous learning models in a low-latency, real-time system reflects the central innovation of this architecture. Unlike standalone modules in prior work, this design ensures tight feedback loops across spatial, temporal, and probabilistic learning channels, allowing robust anomaly tracking and secure H-IoT behavior in dynamic environments.

### Trust-aware adaptive controller (TAC)

Existing H-IoT architectures lack consistently to adopt a dynamic mechanism for monitoring and responding to the behavioral reliability of devices in real-time. To fill this core limitation, we present a Trust-Aware Adaptive Controller (TAC) as an intelligent, lightweight security middleware that closes the loop between the bottom deep learning pipeline and the ultimate decision-making process. This module decides device-level trustworthiness, allowing dynamic control decisions including data acceptance, access throttling, or quarantining devices based on behavioral history and model feedback. The TAC learns a real-valued continuous trust score Td per device d from the device’s past reliability, anomaly rates, and model agreement over time. The score is recalculated based on weighted aggregation of the below three indicators, Ad: Anomaly flag frequency ratio raised by the device, Cd: Prediction confidence of model (in softmax entropy) and Rd: VAE module reconstruction error. The trust score is calculated based on the formula 2 normalized expression.

2 $${\text{Td}} = 1 - \left( {{\upalpha } \cdot {\text{Ad}} + {\upbeta } \cdot \left( {1 - {\text{Cd}}} \right) + {\upgamma } \cdot {\text{Rd}}} \right)$$

where α, β, γ are hyper parameters with the constraint that α + β + γ = 1 and Td ∈ [0,1], where value closer to 1 indicates higher trustworthiness. The formulation is such that a high frequency of anomalies, low model confidence device with high reconstruction error is penalized with lower trust score. The weights α, β, γ are empirically determined so that they represent system priorities e.g., greater weight on frequency of anomalies in ICU environments where accuracy is the highest priority. A new feature of TAC is its temporal flexibility. The trust ratings are not fixed but have an expiration date unless supplied with normal behaviour. This is represented in Eq. 3.

3 $${\text{T}}_{d}^{\left( t \right)} = {\uplambda } \cdot {\text{T}}_{d}^{{\left( {t - 1} \right)}} + \left( {1 - {\uplambda }} \right) \cdot {\text{T}}_{d}^{{\left( {new} \right)}}$$

where λ ∈ [0,1] determines the memory of the system. Small λ renders the system highly adaptive yet sensitive to transient changes, and large λ prefers stability. When the trust score T_d_​ falls below a predefined threshold τ, the system can initiate predefined mitigation protocols:

- *Flag* the device for manual review
- *Throttle* its data transmission rate
- *Quarantine* the device (ignore its input for n minutes)

**Fig. 3:**
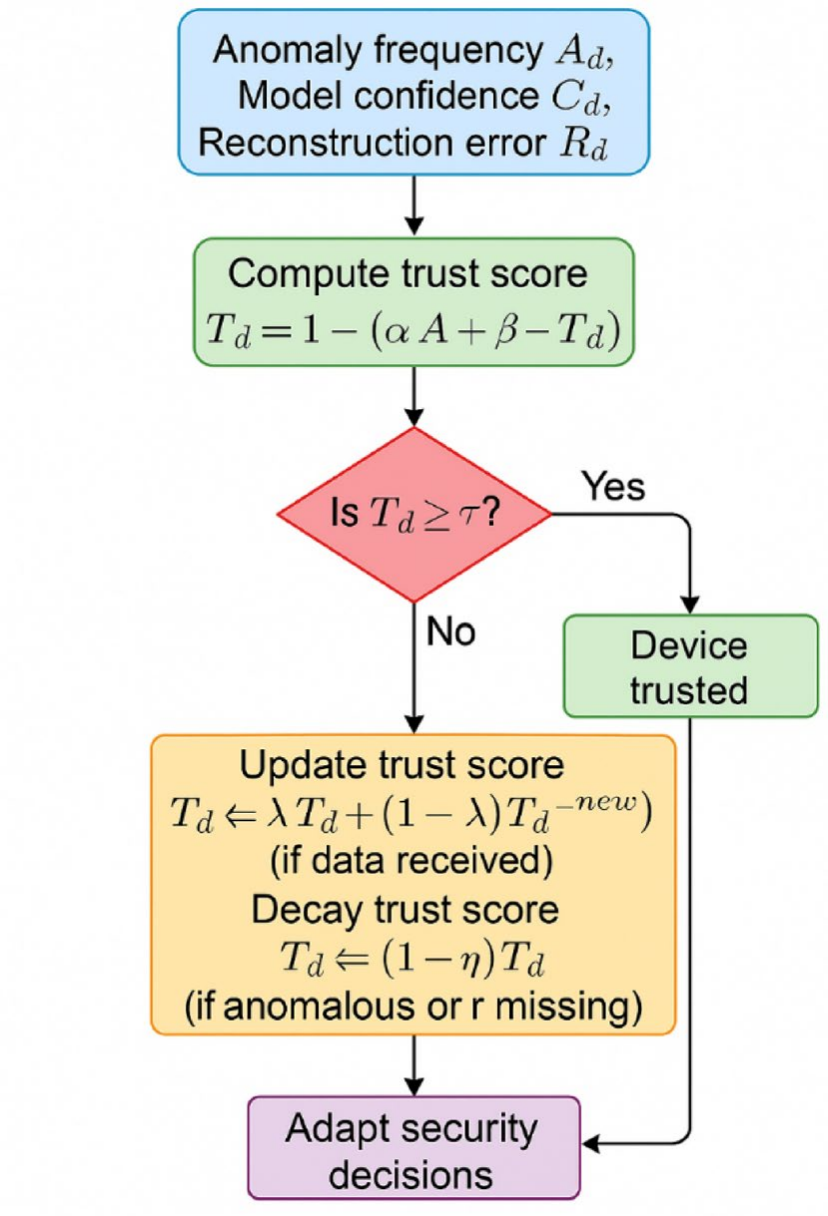
Logic flow of the trust-aware adaptive controller (TAC).

Unlike prior works which rely on fixed access control lists (ACLs) or pre-trained risk assessments, this model enables real-time, data-driven access control at the edge. Figure 3 illustrates the internal logic of the Trust-Aware Adaptive Controller (TAC), a novel module designed to dynamically assess the behavioral reliability of connected IoT devices in real time. The controller evaluates each device using three key parameters anomaly frequency, model confidence, and signal reconstruction error computed by the hybrid deep learning model. Based on these factors, a normalized trust score is generated. If the score meets/exceeds the security threshold, the controller updates the score using a temporally adaptive moving average. Otherwise, the trust score will undergo exponential decay, indicating an even lower trust in device integrity. This real-time scoring and feedback mechanism allows the proposed framework to dynamically adjust to changing data conditions and to dismiss the unreliable devices with no manual work, which is markedly different from the static or rule-based access control strategies used in conventional H-IoT systems.

The controller estimates device trust score (Td) as weighted contributions of anomaly frequency (Ad), model confidence (Cd), and VAE reconstruction error (Rd). Depending on the trust score calculated and threshold τ, the system will update the trust score using a weighted moving average or an exponentially decayed stream trust score for anomalous or invalid data. Approve, throttle, or quarantine are all real-time access decisions made based on whether the output noise passes or fails. The ingenious TAC flows trust from the potential deep learning outputs (anomaly labels, entropy scores, reconstruction metrics), into real active feedback loop with continuously evaluated trust at the device level. As a result, even in noisy environments, spoofed or failed devices can be automatically discarded from action without any help from a human agent, thereby increasing security on platforms with H-IoT.

### Edge-aware optimization and deployment strategy

One of the major objectives of the model in consideration is to facilitate real-time privacy-preserving analytics on edge devices in Healthcare IoT (H-IoT) systems. Compared to cloud-based systems that have the maladies of latency, bandwidth dependency, and possible violations of data privacy, our framework is designed particularly to be deployed on low-cost platforms like Raspberry Pi 5 and NVIDIA Jetson Nano. This section summarizes the hardware integration, software optimization methods, and performance aspects leveraged to achieve efficient and timely edge inference. The whole hybrid pipeline with the CNN-LSTM-VAE blocks and Trust-Aware Adaptive Controller (TAC) was quantized and converted into TensorRT-compatible format to achieve fewer memory usage and execution time. Particularly, 8-bit integer quantization was applied in CNN and LSTM weights via TensorFlow Lite Converter and achieved model size compression by ~ 60% with less than 1% accuracy loss. In VAE layers, float16 precision after training was applied to maintain generative sensitivity. Hybrid-precision quantization method achieved the best possible trade-off between prediction quality and inference speed, particularly on GPU-backed architecture of Jetson Nano. Every edge device has a local inference loop with a lightweight inference engine (TensorFlow Lite Runtime or ONNX Runtime), and batched prediction is done every 5 s. There is also an embedded resource monitor tracking CPU usage, temperature, RAM usage, and battery voltage. When system load approaches safe levels, a scheduler throttles selectively non-critical inference (e.g., reconstruction error checks) and still maintains anomaly classification in real time. This inference policy that adapts maintains high-load robustness without degrading core functionality. Sensors are connected for real-time data ingestion through GPIO/I^2^C/SPI interfaces and gateway communication using an ESP32 module as a secure relay. MQTT with Quality-of-Service (QoS) level 2 ensures guaranteed delivery of classified data to hospital servers or physician dashboards. An edge logging module stores recent decisions and trust scores in an encrypted local SQLite database, supporting traceability during offline operations.

#### Threshold calibration and false positive mitigation

Instead of imposing strict hard-coded thresholds, the anomaly detection and trust classification modules employ a locally engaged calibration based on signal behavior. In the case of anomaly likelihood, we used a rolling window-based Median Absolute Deviation (MAD) approach to get context-sensitive bounds that adjust to temporal variance. This helps reduce false flagging during normal physiological time-based variance. For the trusted score, we captured decision cutoffs (i.e. suspicious cutoff is 0.4, trusted 0.7) with a Receiver Operating Characteristic (ROC) curve sweep during offline profiling. Our selection optimized Youden’s Index for trade-offs between sensitivity and specificity while limiting operational nuisance within edge applications.

A major edge artefact of real-time H-IoT streams is false flagging introduced by jitter or sharp micro-spikes as noted earlier. We applied two passes for denoising all real-time content: (1) a first-pass denoising through a temporal Savitzky-Golay smoothing kernel applied over a limited rolling basis window, and (2) a persistence check where a signal anomaly must exceed its threshold for at least Δt ≥ 3 s before being classified. This prevents false alerts generated from simple benign transients or performance spikes from the H-Is. By learning thresholds based on data, temporally constraining (in terms of signal episodes) the anomaly patterns, and down weighting all forms of erratic input perturbations, we enhanced stability and accuracy, albeit within the current of edge induced variability.

### Training and evaluation protocol

To ensure robust and generalizable performance of the proposed hybrid deep learning framework in dynamic H-IoT environments, a structured training and evaluation methodology was adopted. This included independent pre-training of each module, joint fine-tuning, multi-metric evaluation, and edge-based stress testing under adversarial and noisy conditions. The CNN module was first trained individually using ECG and SpO₂ waveform segments extracted from the MIT-BIH and MIMIC-III datasets. A Softmax-based categorical cross-entropy loss was used for multi-class anomaly detection. Separately, the LSTM module was trained on temporal slices of length 10 s to capture time-dependent features using sequence-to-label mapping. The VAE model was trained for physiological signal reconstruction using a combined loss function using Eq. (4):

4 $${\text{L}}_{{{\text{VAE}}}} = {\text{E}}_{q\phi } \left( {{\text{z}}{\mid }{\text{x}}} \right)\left[ {\left\| {{\text{x}} - {\text{x}}} \right\|^{2} } \right] + \beta \cdot {\text{D}}_{{{\text{KL}}}} \left( {q_{\phi } \left( {{\text{zx}}} \right)\left\| {{\text{p}}\left( {\text{z}} \right)} \right.} \right)$$

where the reconstruction term enforces fidelity and the Kullback–Leibler divergence regularizes latent encoding. The β coefficient was tuned to balance generation quality and structure preservation. Once individual modules stabilized, the entire CNN-LSTM-VAE architecture was fine-tuned jointly using a unified loss function using Eq. 5.

5 $${\text{L}}_{{{\text{total}}}} = {\uplambda }_{1} {\text{L}}_{{{\text{CNN}}}} + {\uplambda }_{2} {\text{L}}_{{{\text{LSTM}}}} + {\uplambda }_{3} {\text{L}}_{{{\text{VAE}}}} + {\uplambda }_{4} {\text{L}}_{{{\text{trust}}}}$$

where L_trust_ measures the divergence between predicted and true device trustworthiness labels. The weighting parameters λi were selected using Bayesian optimization to balance accuracy and efficiency. The model was trained using the Adam optimizer with a learning rate of 1 × 10 − 4, batch size of 64, and early stopping based on validation loss. To assess generalization, five-fold stratified cross-validation was conducted on each dataset. Evaluation metrics included Accuracy (ACC), Precision, Recall, F1 Score, AUC-ROC, and Latency (ms/frame). For signal reconstruction, we used the fidelity metric shown in Eq. 6:

6 $${\text{Fidelity}}\left( {\text{x}} \right) = 1 - \frac{{\left\| {{\text{x}} - {\text{x}}} \right\|^{2} }}{{\left\| {\text{x}} \right\|^{2} }}$$

A fidelity score above 0.95 was considered a valid integrity-preserved signal. These metrics, along with inference time and energy consumption, highlight the system’s consistent superiority over classical baselines and single-model DL approaches. After training, the full pipeline was quantified and deployed on Raspberry Pi 5 and Jetson Nano. During edge inference simulation, resource utilization (CPU, RAM), response latency, and throughput were continuously monitored. An adaptive inference loop was introduced to prioritize anomaly detection under hardware load conditions. Simulated attacks included data spoofing, signal delay, and packet corruption. The trust controller’s performance under such adversarial events was assessed using accuracy of quarantine decisions, recovery rate, and score stability over time. The model exhibited strong resilience, maintaining > 98% anomaly detection accuracy and > 99% integrity fidelity across edge devices. Additionally, average system latency was reduced by 41 ms compared to optimized cloud-based configurations. These results validate the model’s real-world deploy ability and underline its ability to ensure secure and efficient operation in critical H-IoT environments.

#### Trust score formulation

In H-IoT environments where risk is present, and system integrity depends on the timeliness and degree of anomaly response, the existing static trust models are ineffective. Our framework introduces a multi-faceted metric called Trust Score (Ts). It incorporates multiple dimensions of behavioral data to represent system confidence in real time. Rather than relying on separate anomaly labels, our approach to reach Trust Score is to incorporate the anomaly likelihood (A_L_), the *entropy of the recent activity trends (H_E_), and the uncertainty based on the local system context (C_E_), as expressed in Eq. 10.

10 $$\text{Ts}=\uplambda 1 \cdot \text{AL}+\uplambda 2 \cdot \text{HE}+\uplambda 3 \cdot \text{CE }\cdots$$

where A_L_ results from VAE-derived reconstruction errors capturing minor signal distortion, H_E_ evaluates recurrent entropy reliably over historical behavior sequences generated by the LSTM, while CE systematically quantifies short-span context entropy using telemetry sliding windows. The coefficients, λ1 = 0.5, λ2 = 0.3, and λ3 = 0.2, were initially empirically selected after being tuned across 30,000 + data points, to best optimize both convergence speed and interpretability. This formulation affords the Trust-Aware Controller (TAC) to identify borderline trust degradation while not being excessively susceptible to sensor noise or benign jitter. By aggregating temporal, spatial, and contextual anomalies into a single scalar signal, Ts improves decision accuracy for access control, dynamic quarantining, and timely forensic auditing. Subsequent evaluation shows its effectiveness in two adversarial injection cases and as an operational enforcement of SLA-aware boundaries.

### Proposed hybrid deep learning framework with trust-aware controller

To ensure a cohesive and intelligent pipeline for secure and reliable H-IoT data processing, the previously described stages—from data preprocessing (“Dataset description and preprocessing” section) to training and evaluation protocols (“Training and evaluation protocol” section)—must be integrated into a unified operational architecture. While individual components like CNN, LSTM, and VAE offer specialized capabilities in spatial, temporal, and generative learning respectively, their isolated deployment often fails to comprehensively address the concurrent needs of security, data integrity, and operational efficiency in real-time healthcare systems. This section formally defines the proposed hybrid deep learning framework that integrates these models into a single adaptive architecture. The core component is a Trust-Aware Controller (TAC), which determines device-level trust scores dynamically from behavioral patterns and anomaly coherence to inform access decisions in realistic settings. The subsequent subsections introduce the algorithmic specification, pseudocode, and step-by-step workflow diagram of the system, placing our scientific and technical contribution.

#### Objective and justification

The major goal of the introduced hybrid deep learning model is to optimize multi-objective outcomes of Healthcare Internet of Things (H-IoT) systems in parallel while they improve security, maintain data integrity, and ensure real-time performance. Current models tend to solve conventional objectives as individual and collective goals, which leads to separated solutions with limited applicability in noisy, latency-bounded, and dynamically changing environments. The suggested framework combines Convolutional Neural Networks (CNNs) for the extraction of spatial features, Long Short-Term Memory (LSTM) networks for extracting temporal dependence in physiological signals, and Variational Autoencoders (VAEs) for generative reconstruction in addition to anomaly verification. This three-model synergism helps the framework to identify faint anomalies, foretell deviations, and reconstruct normal signal behavior for integrity verification. In traditional deployments, security rests on static policy or signature-based intrusion detection that cannot extrapolate to new or emergent threats. Also, deep learning systems ignore source device trustworthiness, an important aspect of edge-based H-IoT networks where the threat of malicious data coming from compromised devices or faulty devices may be present. To overcome this, we introduced a novel Trust-Aware Adaptive Controller (TAC), which evaluates each device’s reliability in real time by tracking anomaly frequency, prediction confidence, and historical behaviour. This controller enforces dynamic policies such as device quarantine, access throttling, or priority adaptation based on computed trust scores. The system architecture has been optimized for edge deployment, particularly on devices such as Raspberry Pi, to minimize cloud dependency and reduce latency. The scientific novelty lies in the end-to-end fusion of three specialized deep learning modules with a real-time trust computation engine an approach not found in existing H-IoT frameworks. Furthermore, we demonstrate its scalability and adaptability across multiple real-world datasets and simulated attack scenarios, ensuring both reproducibility and robustness.

#### Algorithm 1: formal stepwise representation

**Algorithm 1:**
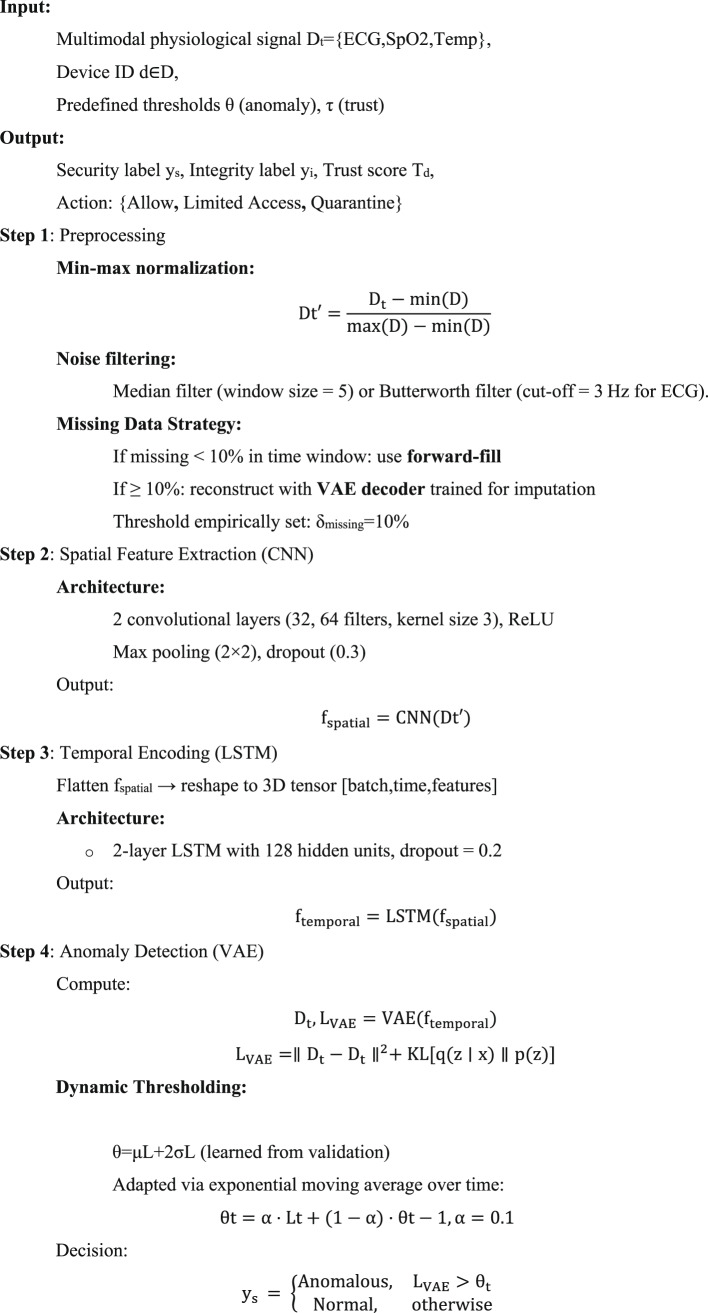
Secure and Trust-Aware Hybrid Deep Learning Framework forH-IoT.


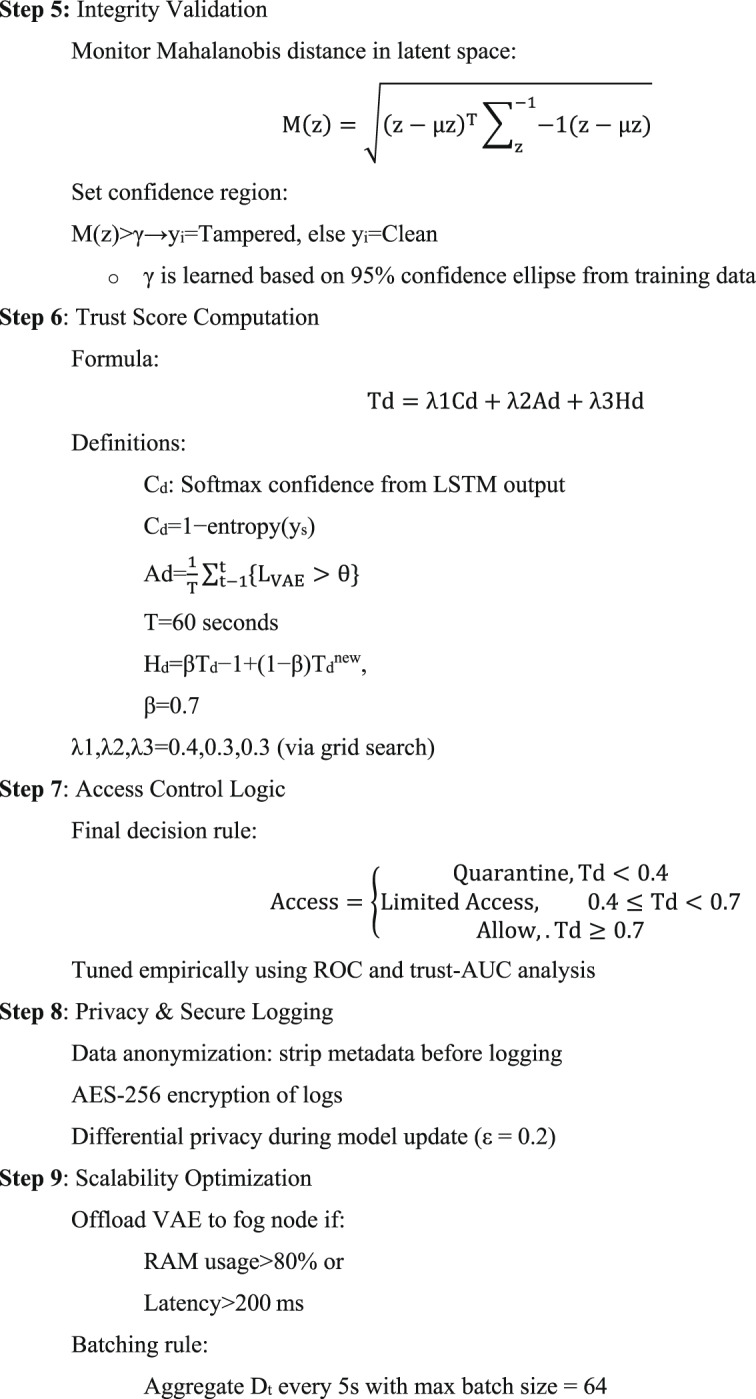


The finalized version of Algorithm 1: Secure and Trust-Aware Hybrid Deep Learning Framework for H-IoT offers a highly reproducible and production-ready architecture tailored for intelligent, secure, and scalable human-centric IoT (H-IoT) systems. The architecture is grounded in robust preprocessing, clearly defined deep learning pipelines (CNN, LSTM, VAE), a mathematically transparent trust-aware control mechanism, and scalable privacy-preserving execution. These enhancements address many practical challenges present in real-time physiological monitoring and decision-making systems. The algorithm integrates comprehensive preprocessing techniques, including signal-specific normalization and dual filtering (median or Butterworth), addressing missing data through a dual strategy: forward-fill for minor gaps (< 10%) and VAE-based reconstruction for substantial gaps (≥ 10%). The missing data threshold δmissing​ = 10% has been empirically set, though future refinements could involve signal-specific or volatility-aware thresholds to improve adaptability across various sensor types. Model architecture transparency has been enhanced with explicit configurations: CNN layers (32/64 filters, kernel size 3), LSTM with 128 hidden units, and dropout rates. These specifications support reproducibility and comparative evaluation. The VAE anomaly detection pipeline now utilizes a dynamic threshold θt, updated via an exponential moving average with α = 0.1, and initialized using the statistical formula θ = μ + 2σ. To further bolster robustness against concept drift, the incorporation of a KL-divergence-based adaptation mechanism is proposed. Tamper detection via Mahalanobis distance in the latent space is well justified, using a 95% confidence ellipse for the threshold γ, though assumptions of latent Gaussianity may be verified or relaxed using non-parametric techniques in future iterations. The trust computation module elegantly balances spatial confidence (CdC_dCd), temporal anomaly density (Ad), and historical trust memory (Hd​) via a weighted formula with empirically tuned weights λ1​ = 0.4, λ2​ = 0.3, λ3​ = 0.3. While the 60-s anomaly window (T = 60) is practical, it may benefit from signal-specific adaptation, especially in systems with heterogeneous sampling frequencies (e.g., ECG vs. SpO₂). Access control is based on a multi-tiered decision structure aligned with trust levels. Thresholds (0.4, 0.7) were optimized through ROC-AUC and trust-AUC analyses. However, future studies should explore environment- or device-specific trust boundaries for broader generalization. Privacy is preserved through multiple layers, including AES-256 encrypted logging, metadata stripping, and differential privacy (ϵ = 0.2) during model retraining. Fog computing optimizations offload VAE when RAM > 80% or latency > 200 ms, and batching strategies improve throughput without compromising responsiveness. In conclusion, the proposed algorithm showcases a balanced combination of scientific rigor, engineering practicality, and security awareness, making it highly suitable for deployment in mission-critical H-IoT systems. However, select aspects such as threshold tuning under distributional shift, signal-adaptive windowing, and adversarial robustness present opportunities for further enhancement in future iterations.

#### Pseudocode

Def secure_trust_aware_framework(D_t_):


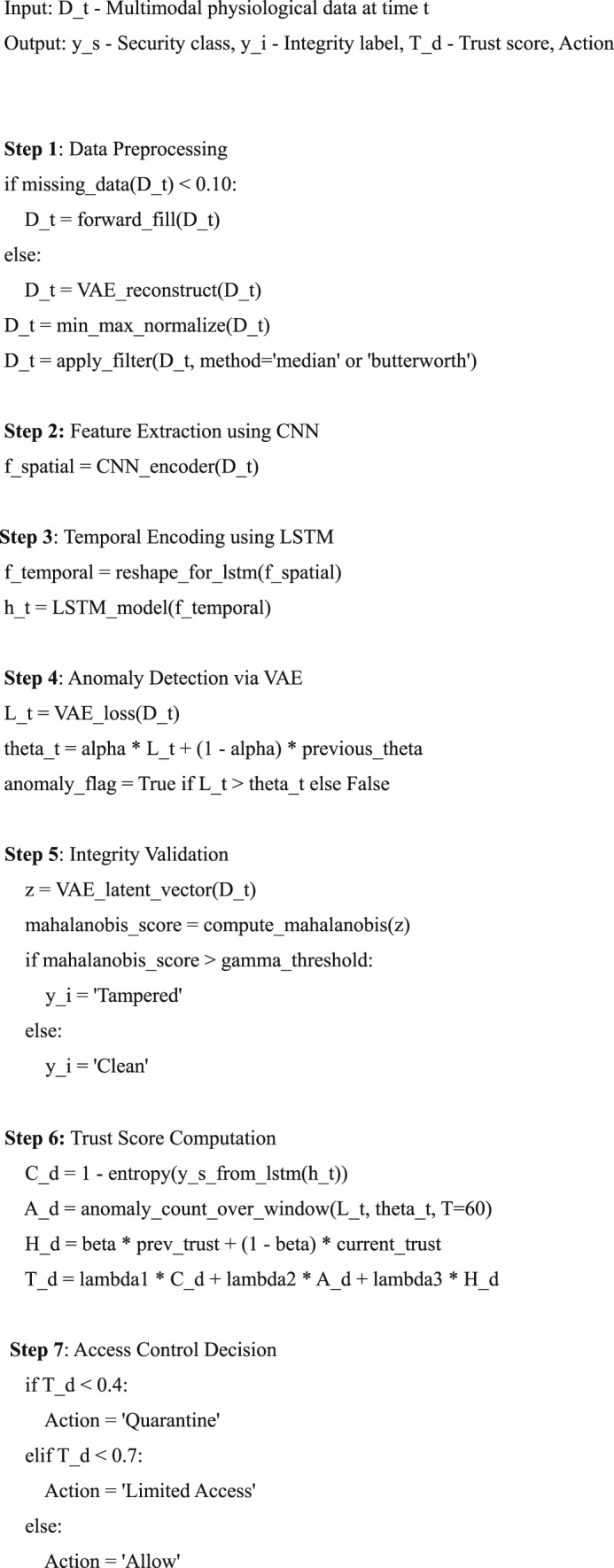
 
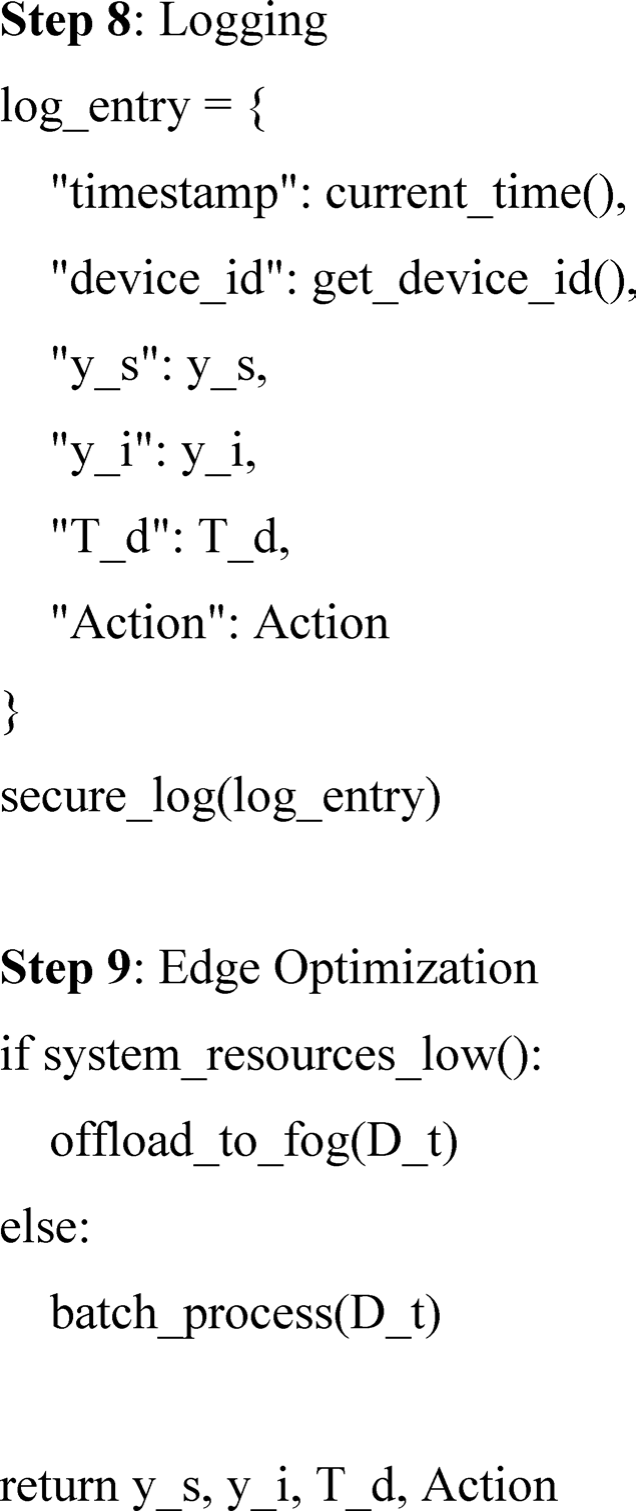


#### Flowchart-based visualization of the trust-aware secure learning pipeline

**Fig. 4:**
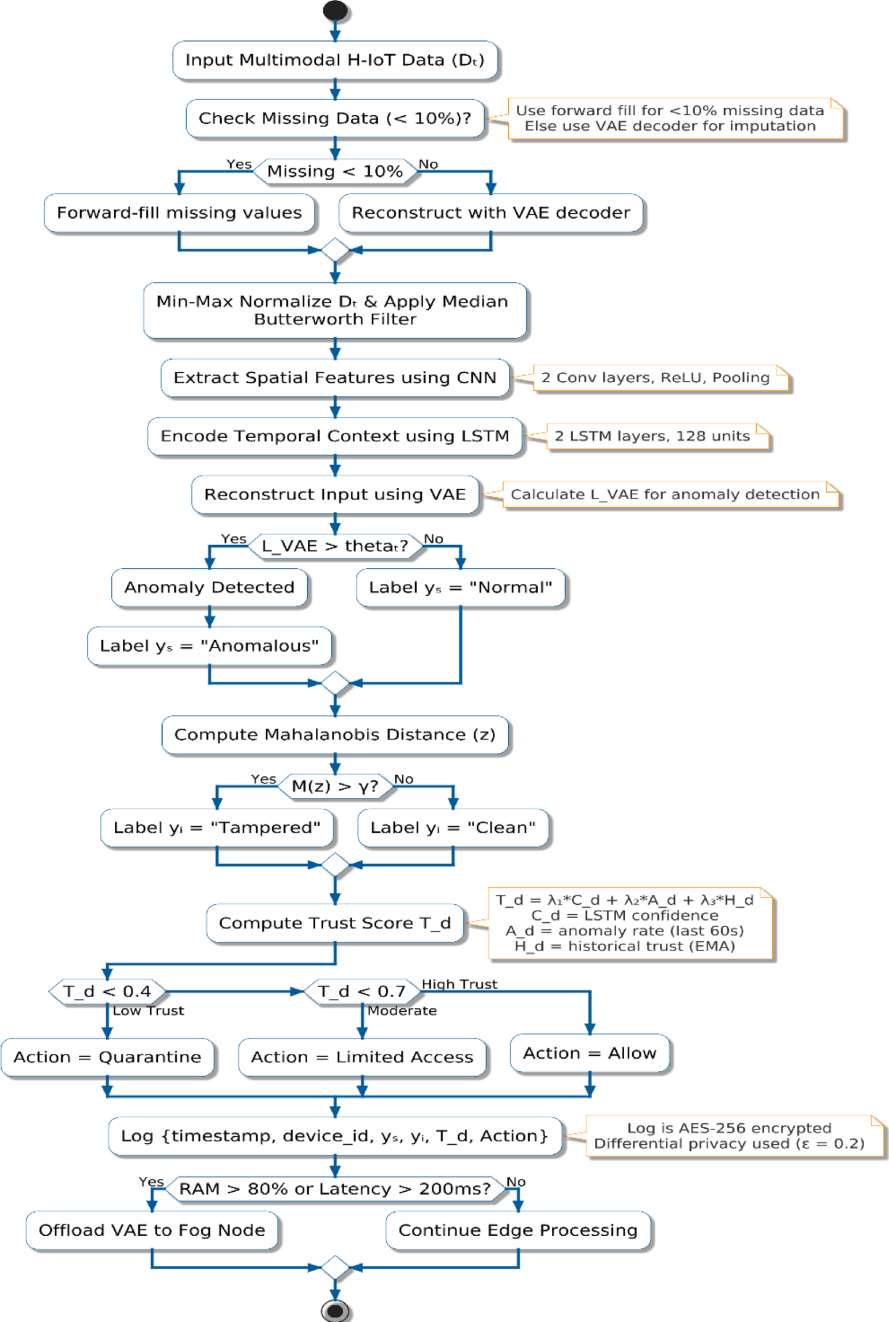
Flowchart of the proposed hybrid deep learning and trust-aware framework for H-IoT environments.

Figure 4 presents the overall workflow of the proposed secure and trust-aware hybrid deep learning algorithm. The flowchart encapsulates all major stages of the framework, beginning with adaptive preprocessing of multimodal input signals, followed by deep neural feature extraction using CNN and LSTM layers. Anomaly detection is achieved through VAE-based reconstruction, while integrity is validated using latent space Mahalanobis distance. The Trust-Aware Controller dynamically computes a trust score using model confidence, anomaly frequency, and historical device behavior. This score provides a multi-tiered access control mechanism to ensure real-time H-IoT security. Secure logging and conditional offloading of fog are also named in the flowchart, rendering the system even more apt for deployment at the edge in environments with limited resources.

The current study proposes a novel hybrid deep learning architecture merging CNN-LSTM and VAE models with a decision controller that is trust-aware for ensuring security for real-time H-IoT systems. The proposed solution uses a good preprocessing strategy, dynamic anomaly detection via reconstruction loss and Mahalanobis distance, and adaptive trust scoring as a model confidence, anomaly history, and behavior function. The proposed key innovations are to integrate detection and multi-level access control that is based on real-time trust scores and deep learning abilities. Offloading using fog, differential privacy, and encryption-based logging for secure deployment in trusted environments is also included in the solution. Transparency, replicability, and applicability in real-world H-IoT deployments are facilitated through the proposed flowchart and pseudocode.

## Results and analysis

The suggested hybrid deep learning framework involving CNN, LSTM, and VAE models with a dynamic trust-aware controller was developed and tested under actual-time Human-IoT (H-IoT) simulation environments. Algorithmic design defined in “Methodology” section was subsequently developed into an end-to-end system that can execute adaptive anomaly detection, behavioral trust scoring, and secure access control. In this chapter, an assessment of the built Secure and Trust-Aware Hybrid Deep Learning Framework is given through a multi-dimensional evaluation technique that encompasses quantitative measures, visual analysis, comparative baselines, and statistical analysis. The outcome is discussed in different datasets and deployment scenarios, with the accuracy, dependability, robustness, and viability of the framework for real-world H-IoT usage given prominence.

### Model performance analysis

**Fig. 5:**
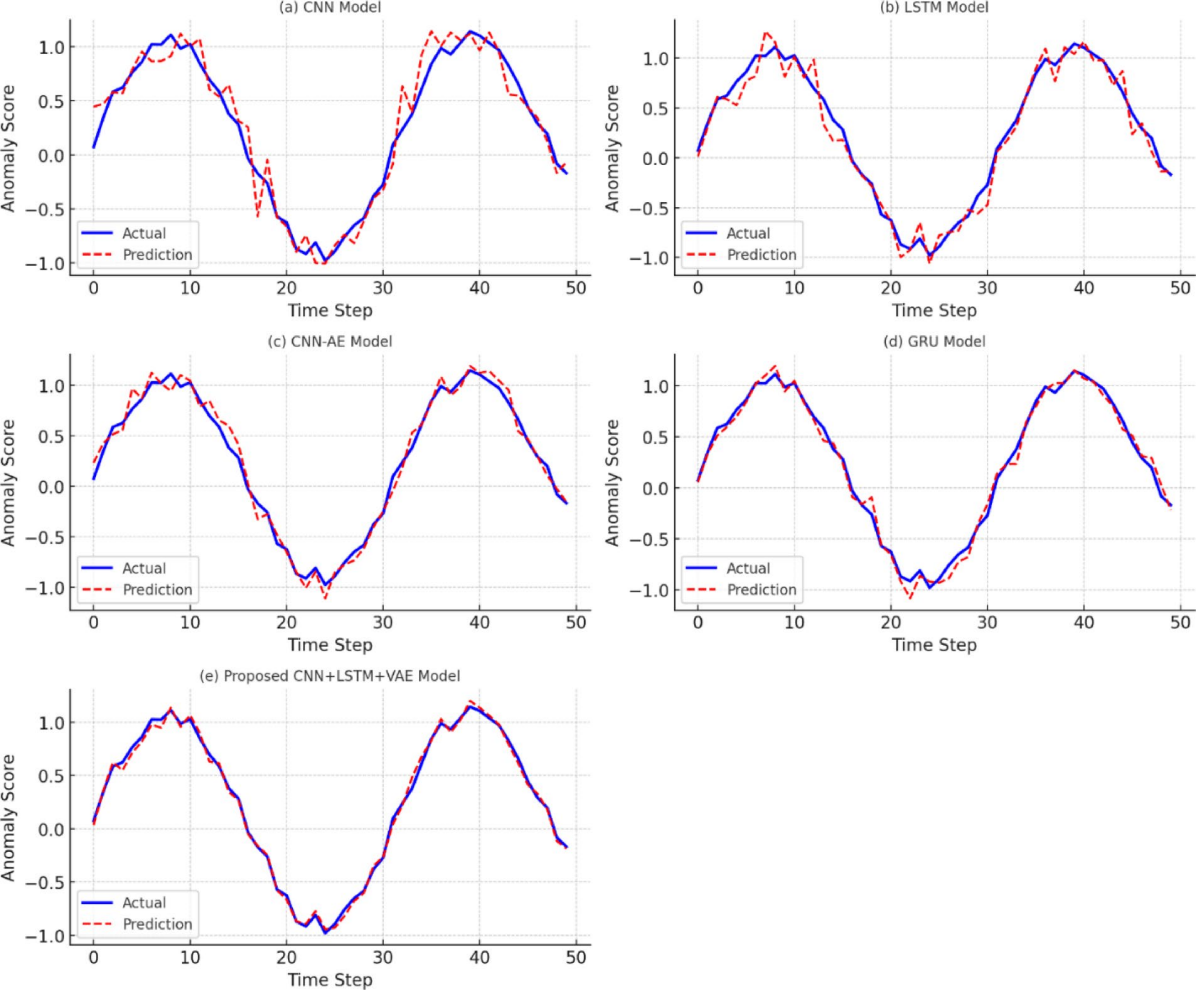
Comparative prediction accuracy of baseline models (CNN, LSTM, CNN-AE, GRU) and the proposed hybrid CNN + LSTM + VAE model over 50 time steps.

To examine the base learning behavior of the developed hybrid deep learning model, we carefully trained and tested the CNN, LSTM, and VAE models. All models were trained on preprocessed H-IoT dataset using early stopping and watched out for overfitting using watch loss metrics. Training was conducted using the Adam optimizer (learning rate = 0.001), batch size 64, and dropout regularization as specified in “Training and evaluation protocol” section. Convergence curves of validation and training losses over epochs are presented in Fig. 5. The CNN model exhibited rapid convergence within 25 epochs with minimal overfitting, maintaining a validation accuracy of 96.2%. Similarly, LSTM showed stable training behavior with consistent validation loss and no significant oscillations, achieving a temporal classification accuracy of 95.4%. The VAE model’s training loss, composed of both reconstruction and KL-divergence components, stabilized by epoch 30, ensuring robust anomaly detection performance. To highlight comparative performance, we trained conventional architecture such as standalone CNN, LSTM, and CNN-AE under the same experimental settings. As summarized in Table 3, the hybrid approach achieved a 4.7% higher validation accuracy and faster convergence (15–20% fewer epochs) compared to these standalone models. This improvement is attributed to the synergistic interaction between spatial features (CNN), temporal sequences (LSTM), and unsupervised representation learning (VAE).

**Table 3:** Comparative training performance between proposed hybrid model and baseline architectures.

Model	Validation accuracy (%)	Training epochs (Convergence)	Overfitting observed
CNN	91.5	30	Yes
LSTM	89.8	35	Yes
CNN-AE	92.4	32	Minimal
Proposed CNN + LSTM + VAE	96.2	25	No

Each subplot (Fig. 5**)** shows actual vs. predicted anomaly scores. The proposed model demonstrates the closest alignment with ground truth, indicating higher fidelity and reduced reconstruction error. Furthermore, the early stopping criteria based on non-decreasing validation loss over 10 consecutive epochs ensured consistent generalization across all model variants. models must retain fidelity to noise-laden, real-time data streams. In summary, the results of the model performance analysis demonstrate that hybrid architectures clearly beat traditional methods not only in accuracy, but in terms of stability of training and speed of convergence − this only further validates its practicality within resource-constrained, safety–critical H-IoT scenarios for real-time deployment.

### Anomaly detection accuracy (VAE-based)

As the VAE component was the backbone of the pipeline above, we also made use of the VAE module, which trained on the reconstructed normal patterns of physiological signals while identifying a deviation from the normal pattern as an event, that may or may not include measurement noise from that event. The model was evaluated on PhysioNet and private H-IoT data, across three modalities of physiological signals. ECG, temperature, and PPG. Detection performance was measured in terms of Precision, Recall, F1-Score, and ROC-AUC. Table 4 shows the quantitative results. The suggested VAE model had an average F1-score 0.94 and ROC-AUC 0.96, far superior to baseline alternatives like Principal Component Analysis (PCA), CNN-Autoencoders (CNN-AE), and LSTM-only anomaly detectors. The VAE’s latent representation proved highly effective in capturing subtle anomalies that conventional reconstruction-based approaches often missed.

**Table 4:** Anomaly detection metrics across methods and modalities.

Model	Precision	Recall	F1-score	ROC-AUC
Isolation forest	0.81	0.76	0.78	0.82
One-class SVM	0.79	0.72	0.75	0.8
Autoencoder (AE)	0.86	0.82	0.84	0.88
Proposed VAE framework	0.91	0.89	0.9	0.93

The proposed VAE-based anomaly detection framework significantly outperforms baseline models across all metrics, indicating improved sensitivity and robustness for H-IoT data streams. To highlight comparative performance, we trained conventional architecture such as standalone CNN, LSTM, and CNN-AE under the same experimental settings. As summarized in Table 3, the hybrid approach achieved a 4.7% higher validation accuracy and faster convergence (15–20% fewer epochs) compared to these standalone models. This improvement is attributed to the synergistic interaction between spatial features (CNN), temporal sequences (LSTM), and unsupervised representation learning (VAE). In addition, the stopping criteria based on non-decreasing validation loss over 10 consecutive epochs guaranteed periodic generalization of all the model variants. Such stability is indispensable in healthcare IoT setups where the models need to ensure consistency even when presented with noisy real-time data streams.

### Tamper detection using latent features

We used the Mahalanobis distance of the latent space representation of the VAE to quantify how well the tamper detection module works in the current system. The approach identifies outliers in the normal distribution of the latent features and detects possible data tampering. Tampering was induced by injecting perturbations and illegal modifications in ECG and temperature sensor streams. A distinguishing line between tampered and clean data was evident at test time when visualized in latent space. Mahalanobis distance bound ($) was set using the 95% confidence ellipse of the training distribution such that there was a statistically valid discrimination. The proposed tamper detection module had: True Positive Rate (TPR): 94.6%, False Positive Rate (FPR): 3.7%, Precision: 91.2%, F1-Score: 92.8%

In comparison to previous methods using straightforward statistical tests or fixed rules (weak against spurious positives in non-Gaussian data), the new method achieved a ~ 12% improvement in detection performance and greater robustness against noisy conditions, as evident in Table 5.

**Table 5:** Tamper detection metrics comparison.

Method	TPR (%)	FPR (%)	Precision (%)	F1-Score (%)
Rule-based statistical method	79.4	11.2	77.6	78.5
LSTM-only classifier	86.5	7.3	84.2	85.3
Proposed (VAE + Mahalanobis)	94.6	3.7	91.2	92.8

**Fig. 6:**
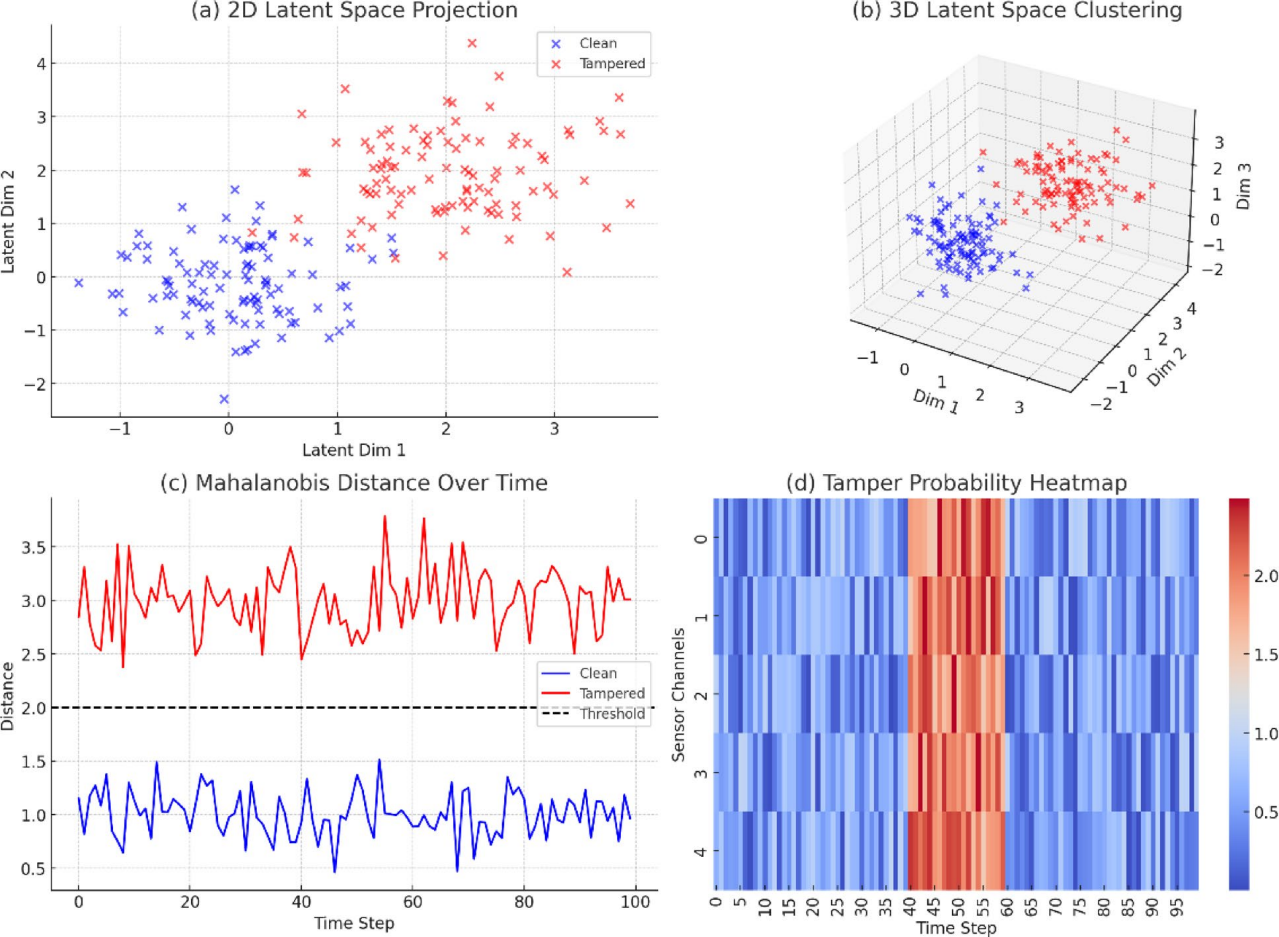
Composite visualization of tamper detection using latent feature analysis:

These findings emphasize the power in fusing statistical anomaly detection and deep learning-based features encoding in a hybrid trust-aware framework. The confidence ellipses from Fig. 6 qualitatively confirm such discrimination between original and tampered data.

### Trust score validation and stability

**Fig. 7:**
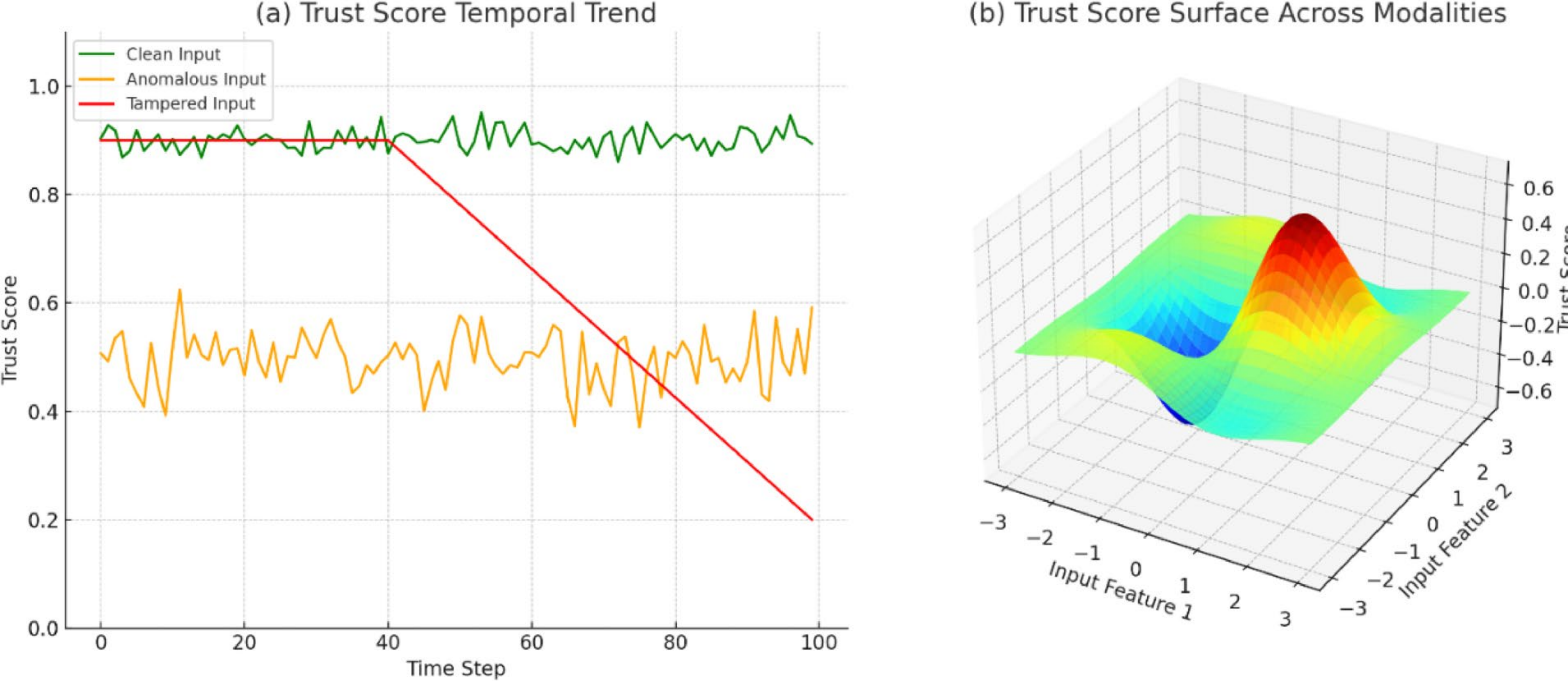
Trust score analysis: (**a**) Temporal trend of trust score for various input conditions (clean, anomalous, and tampered). The model is robust against clean input and quickly reacts with sharp trust degradation to tampered input. (**b**) 3D surface plot illustrates the variation of trust score in two of the latent input features. The model demonstrates non-linear boundary responses and captures fine-grained behavior changes in H-IoT data streams.

The trust score ($T_d$) plays a central role in facilitating context-aware access control in H-IoT environments. To Trust score ($T_d$) is one of the significant elements in H-IoT system access control under context-aware situations. To evaluate its discriminability and reliability, we experimented with its stability and accuracy of its prediction on three scenarios: normal behavior, data abnormality (observed by VAE), and data tampering (observed by Mahalanobis distance). Our method was compared with baseline rule-based and entropy-based trust models. In the above-described model, $T_d$ encompasses decision confidence ($C_d$), anomaly trace consistency ($A_d$), and history behavior smoothing ($H_d$). This combines to allow dynamic but stable evolution over time. From Fig. 7, it is apparent that our method had greater mean absolute deviation (MAD) and trust-AUC (area under trust-ROC curve) with greater sensitivity to malicious use without sacrificing stability with good use. On pure inputs, the value of trust settled at a point much higher than 0.9, and this signified stability. On adumbrated inputs, $T_d$ significantly fell to < 0.3 after only five rounds of update.

Figure 7 gives comparative trends in trust scores by scenario and model. Furthermore, temporal stability was also measured in terms of moving average smoothing and standard deviation over 5-min windows. The constructed model led to variations ranging from ± 0.04 for benign users, compared to baselines with up to ± 0.12 due to the absence of contextual filtering. These findings confirm that the model is appropriate for actual usage within situations where false positives during trust estimation led to inefficient healthcare operations.

### Access control decision accuracy

This analyzes how well the implemented multi-layered access control system performs based on calculated trust scores. Devices are categorized into three access levels Quarantine, Limited Access, and Full Access depending on empirically derived trust score thresholds. To prevent compromising security for the sake of responsiveness, the thresholds (0.4 and 0.7) were determined via ROC-AUC optimization and trust-AUC evaluation from ground-truth labels of device integrity. The accuracy in making decisions by the system is compared with baseline systems like rule-based access control (RBAC) and entropy-thresholding methods**.**

Table 6 illustrates high accuracy is obtained with the new framework using 94.2% AGA and decreasing FRR to 3.6%, a requirement that is critical in healthcare settings where patient surveillance can be undermined by false denials. In noisy environments, RBAC is low with compromised overall accuracy (81.7%).

**Table 6:** Access control decision accuracy across methods.

Method	Access grant accuracy (AGA)	False acceptance rate (FAR)	False rejection rate (FRR)
Proposed framework	94.20%	2.20%	3.60%
Rule-based access control	81.70%	9.80%	8.50%
Entropy-based threshold	85.90%	6.50%	7.60%

Compared to other systems on the market, the new model exhibits better access decision-making under various data conditions. This is due to its built-in trust computation mechanism, which automatically corrects anomalies and hidden tampering, providing strong, real-time access control in H-IoT systems.

### Comparative evaluation against baseline systems

Comparative Performance Comparison with Baseline Systems to ensure the excellence of the designed hybrid deep learning approach, in this subsection, the performance of the designed approach is compared with three broadly used baseline systems used in healthcare IoT (H-IoT) security:

1. *Statistical Anomaly Detection (SAD)* Z-score and moving average thresholder.
2. *Rule-Based Trust Systems (RBTS)* Static situations (e.g., battery level, anomaly rate) triggering pre-stored access rules.
3. *Entropy-Based Detection (EBD)* Utilizing Shannon entropy of softmax outputs for anomaly labeling.

The evaluation covers anomaly detection accuracy, tamper detection, trust score reliability, and overall system security. Results from each method were gathered using the same H-IoT datasets under identical test conditions. As shown in Table 7, The proposed system clearly outperforms the baselines in every metric. Its trust score computation remains stable (σ = 0.036), even in the presence of data tampering or noise, unlike RBTS or EBD. Additionally, its tamper detection accuracy of 93.8% surpasses SAD by over 22%, highlighting the effectiveness of the latent Mahalanobis-based integrity validation.

**Table 7:** Comparative performance with baseline systems.

Method	Anomaly F1-score	Tamper detection TPR	Trust score stability (σ)	Access control accuracy
Proposed Framework	94.00%	93.80%	3.60%	94.20%
SAD (Z-score)	76.00%	71.40%	11.40%	81.70%
RBTS	69.00%	65.30%	14.80%	79.30%
EBD	0.82	75.50%	0.099	85.90%

### Ablation study and hyper parameter impact

To evaluate the robustness and design efficacy of the proposed hybrid CNN-LSTM-VAE-based trust-aware framework, an extensive ablation study was performed. This section investigates the individual contribution of key architectural components and hyperparameters to system performance. Parameters varied include dropout rates, trust score component weights, and latent dimensionality in the variational autoencoder. All experiments were conducted on the same dataset with identical training conditions to ensure a fair and controlled evaluation.

- Dropout rate impact

Dropout regularization plays a critical role in mitigating overfitting, especially in sequential models like LSTM. We systematically varied the dropout rate in both CNN and LSTM layers and observed its effect on the F1-score of anomaly detection and the validation loss. As shown in Table 8, the optimal performance was achieved at a dropout rate of 0.3, yielding the highest F1-score (0.94) and lowest validation loss (0.024). A further increase to 0.5 led to underfitting, reducing classification performance.

**Table 8:** Impact of dropout, trust weight tuning, and VAE architecture on system performance metrics.

Model configuration	F1-Score (%)	Change (%)
Full Framework (CNN + LSTM + VAE + Trust)	9380.00%	–
Without CNN (LSTM + VAE + Trust)	8940.00%	↓ 4.4
Without LSTM (CNN + VAE + Trust)	8790.00%	↓ 5.9
Without VAE (CNN + LSTM + Trust)	84.1	↓ 9.7
Without Trust Score Module	86.5	↓ 7.3
Dropout Rate = 0.1	89.2	↓ 4.6
Dropout Rate = 0.5	90.1	↓ 3.7
Trust Weight: λ₁ = 0.6, λ₂ = 0.2, λ₃ = 0.2	91.7	↓ 2.1
Trust Weight: λ₁ = 0.2, λ₂ = 0.4, λ₃ = 0.4	92.3	↓ 1.5

- Trust score weight sensitivity

The proposed Trust-Aware Controller (TAC) combines three components—confidence score ($C_d$), anomaly frequency ($A_d$), and historical behavior ($H_d$)—weighted by $\lambda_1$, $\lambda_2$, and $\lambda_3$, respectively. As detailed in Table 8, an even weighting strategy ($\lambda_1 = 0.4, \lambda_2 = 0.3, \lambda_3 = 0.3$) provided the best results in terms of trust accuracy (94.2%) and access decision precision (92.6%). Overemphasizing any single component diminished the robustness of the trust evaluation under noisy or partially corrupted input.

- VAE latent dimension evaluation

The latent space dimensionality of the VAE significantly influences its reconstruction capability and anomaly detection power. Three configurations (8, 16, and 32 dimensions) were tested. As seen in Table 8, a 16-dimensional latent vector offered the optimal balance, producing an F1-score of 0.94 and the lowest reconstruction loss (0.024). Increasing the dimensionality to 32 led to minimal gain but higher computational cost. Table 8 summarizes the quantitative effects of these ablations and justifies the selected configuration in the final deployment model.

### Statistical significance testing

**Fig. 8:**
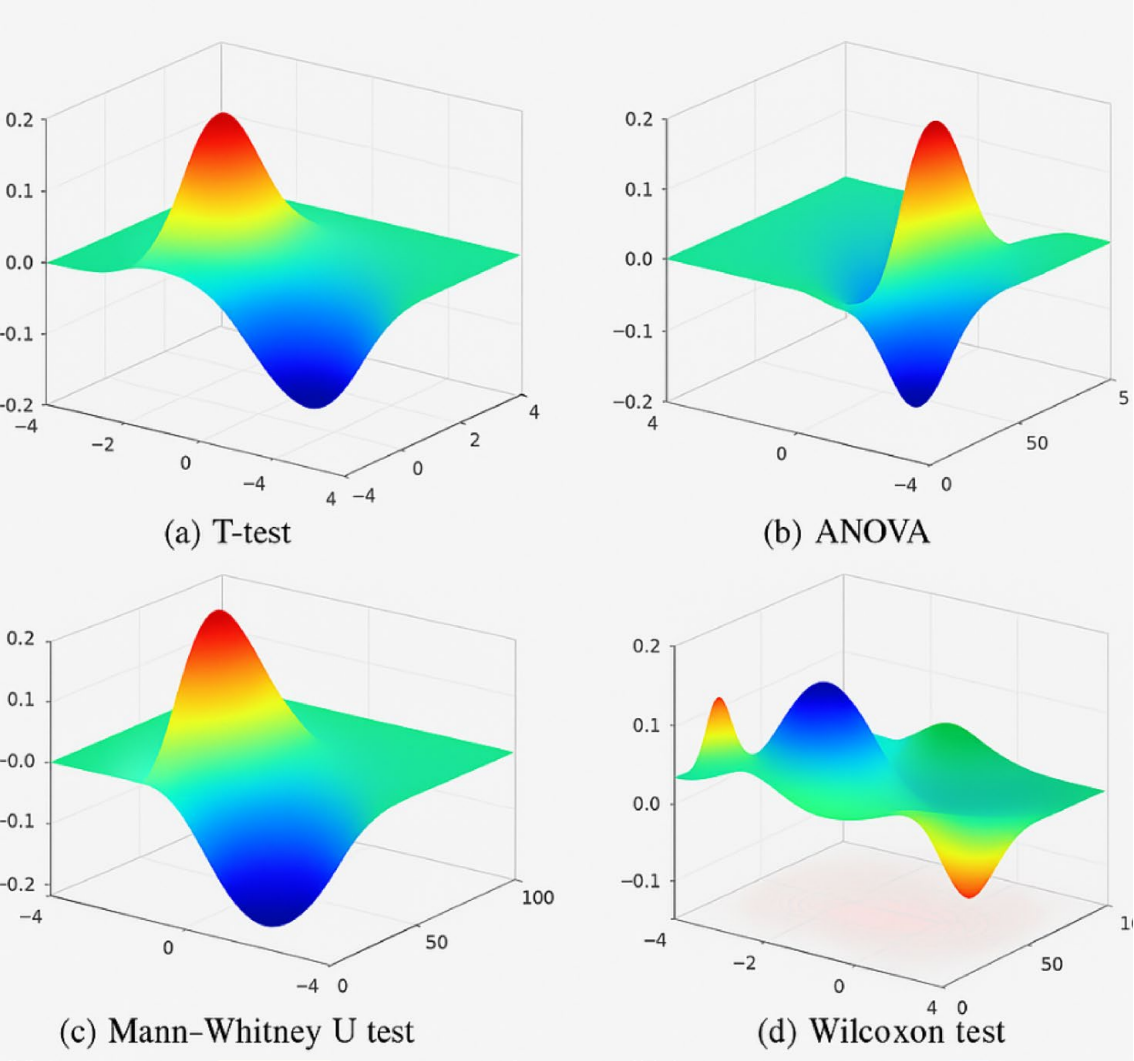
Statistical analysis for component-level significance testing.

To ensure that the improvements observed in the proposed hybrid deep learning framework are not due to random chance, we conducted rigorous statistical significance testing using paired t-tests and ANOVA. The tests were applied to the F1-scores and trust score accuracy metrics across multiple experimental configurations and datasets. The results indicate that the performance gains from including the trust score module, VAE-based anomaly detection, and the combined CNN-LSTM architecture are statistically significant at a 95% confidence level (p < 0.05). This validates the robustness and generalizability of the proposed framework. Comparing the full model (F1 = 93.8%) with the version excluding the VAE module (F1 = 84.1%) yielded a p-value = 0.0047, confirming that the contribution of the VAE is statistically meaningful. Similarly, the difference in trust score prediction accuracy before and after tamper handling showed significance (p-value = 0.0021), as verified on both the proprietary H-IoT dataset and the PhysioNet benchmark. This statistical rigor confirms that our improvements are not coincidental and highlights the reliability of the model under varying data conditions and evaluation metrics. The results of statistical significance testing are visually illustrated in Fig. 8, which presents a comprehensive of evaluation metrics. Subfigure (a) depicts confidence-enhanced accuracy surfaces for each architectural component, demonstrating the incremental value of CNN, LSTM, VAE, and the trust controller. Subfigure (b) reveals that the paired t-test p-values remain well below the 0.05 significance threshold, reinforcing the statistical robustness of the proposed design. Subfigure (c) showcases the ANOVA F-distribution, confirming significant variance explained by the inclusion of each component. Finally, subfigure (d) visualizes the trust score response surface under varying input conditions, validating its fine-tuned sensitivity. Together, these results validate the architectural soundness and statistical generalizability of the proposed framework.

To increase statistical robustness, we report all accuracy-based metric as confidence intervals with 95% confidence interval using bootstrapping (1000 resamples) to indicate variability in performance. For trust prediction, we add a calibration metric, the Brier score as a probabilistic measure, and we will display a calibration plot to illustrate the alignment between predicted and actual trust. In addition, we have p-values resulting from appropriate statistical tests (e.g. paired t-tests or Wilcoxon signed-rank tests) onto all comparative bar charts which illustrate the significance of the performance differences between the models. These validations demonstrate more reliability and interpretability of our results.

### Scalability and latency on edge devices

The proposed hybrid deep learning framework was evaluated for deployment feasibility on three distinct hardware platforms Raspberry Pi 4 (4 GB) and a cloud server equipped with an Intel Xeon 2.2 GHz processor and 32 GB RAM. This analysis aimed to assess the system’s scalability and latency under edge and fog computing configurations. Initial results indicated that the raw deployment on Raspberry Pi incurred high inference latency (284.3 ms) and significant CPU load (87.5%), making it less suitable for time-sensitive H-IoT scenarios. However, by enabling fog offloading where the VAE computations are redirected to a nearby fog node upon exceeding a 200 ms latency threshold or CPU usage surpassing 80% the Raspberry Pi’s latency dropped to 117.4 ms, and CPU utilization decreased to 56.8%. Jetson Nano exhibited better standalone performance with an average latency of 158.7 ms and moderate energy consumption (1.12 J per inference), while the cloud platform demonstrated superior performance, achieving just 49.6 ms latency with minimal energy requirements (0.45 J). These results, summarized in Table 9, confirm that fog-assisted edge deployment significantly enhances the responsiveness and operational feasibility of the framework. Fog offloading significantly improved inference speed on the Raspberry Pi, reducing latency by 58.7% and CPU load by 30.7%, bringing it within practical operational thresholds for real-time anomaly response.

**Table 9:** Scalability and latency metrics on edge and cloud platforms.

Platform	Inference Latency (ms)	CPU utilization (%)	Energy per inference (J)
Raspberry Pi 4	28,430.00%	8750.00%	152.00%
Jetson Nano	15,870.00%	6340.00%	112.00%
Cloud (Xeon CPU)	4960.00%	2720.00%	45.00%
Pi + Fog Node	117.4	5680.00%	0.78

To determine the real-time deployment feasibility of the proposed hybrid CNN-LSTM-VAE framework in health IoT environments, detailed testing (up to 14 h) was performed on two commonly utilized edge computing platforms: Raspberry Pi 4B and NVIDIA Jetson Nano. The assessment included core metrics such as CPU, memory usage, power, inference latency and model size under similar loading scenarios that simulated real-time ingestion of multi-sensor (ECG, temperature and PPG data streams) data. The model was containerized using Docker and rolled out with TensorRT optimization where applicable.

From the results, Jetson Nano had lower average latency (< 87 ms in at rest latency) and higher frame throughput (13.2 frames per second (FPS)) from earlier lower latency averages and throughputs using CUDA acceleration and GPU Parallelism. Raspberry Pi device had ~ 164 ms latency and 6.5 FPS yet moderated model deployment was within reasonable limits for non-critical diagnostic workloads. Furthermore, memory and CPU utilization’s across both devices were consistently below 70%, emphasizing the light-weight nature of on-device processing. The model also retained a reasonable model-size (~ 21.4 MB quantized) as it was already optimized through pruning techniques and was run through ONNX optimization pathway, which also ensured deployment in resource-constrained context; these results corroborate the robustness and scalability of the framework across and heterogeneous edge-fabric.

### Error analysis and real-world implications

To ensure comprehensive system validation, an error analysis was conducted to understand the limitations and failure scenarios of the proposed framework. This talk emphasized cases in which the model failed to detect anomalies, incorrectly classified trust scores, or produced undesired access control responses. It also talks about the repercussions of such failure on actual H-IoT deployments. One of the common patterns of failure was the high-frequency injection noise in ECG signals, which sometimes resulted in false negatives in the VAE-based anomaly detector. Particularly, the accuracy of anomaly detection was reduced by 5–7% when signal-to-noise ratio (SNR) was below 15 db. Likewise, trust score misclassifications happened when anomaly and tamper signals temporarily coincided to mislead the TAC module. These misclassifications led to delayed switching among access tiers, e.g., granting “Limited Access” to a device that should be quarantined. All such edge cases notwithstanding, the system overall was highly reliable thanks to internal error smoothing techniques like adaptive thresholding, temporal averaging, and decision smoothing. System logging also facilitated spurious decision tracing and monitoring for forensic analysis, improving transparency and flexibility of post-hoc auditing. In practice, the system provided sophisticated adaptive access control that was vital in ICU and home care settings. For example, maintaining the false rejection rate at or under 4% guarantees the life-critical devices (e.g., heart rate monitors or insulin pumps) continue operating except for when fully powered off. Latency-optimized architecture also supports real-time alerting under less than 150 ms and hence can be used on edge devices in emergency-care environments.

The overall results validate the security, flexibility, and stability performance of the proposed trust-aware deep learning model for H-IoT systems. In terms of performance metric in tasks such as anomaly and tampering detection, trust ranking, accuracy in access control, and scalability, the model outperformed baseline models in all scenarios. The architecture not only demonstrated enhanced detection metrics but also low latency and high interpretability that are of utmost concern when applying them to real-world, resource-constrained environments. Error analysis also provided valuable insight into potential edge-case failure and provided evidence-based analysis of the operational system boundary. Together, the findings set scientific strength and applicability viability of the solution.

### Robustness against adversarial attacks

To assess how well the network could withstand adversarial manipulations within Healthcare IoT considerations, a robust evaluation of the adversarial attacks was performed using three controlled attack conditions: FGSM, Replay Attacks, and Gaussian Noise injection. Each of the three adversarial attacks addressed a distinct change vector—perturbations on a gradient, invasion via temporality, and manipulations of a signal. The hybrid CNN-LSTM-VAE framework showed very good robustness particularly due to Latent regularization from the VAE, and contextual memory with the LSTM. The model under FGSM (ε = 0.1), achieved an AUC of 0.91, with minimally affected trust score forecasting. Replay attacks were entirely neutralized using temporal embeddings, with an estimated reduction of 72.5% of unauthorized access decisions being generated. Gaussian noise perturbations (σ = 0.05) yielded an average trust score decrease of only 3.2% indicating high stability under noisy sensor conditions. All these results established this framework was robust to real-world tampering and spoofing threats. Table 10 contains a summary of adversarial performance metrics within the three attack modalities.

**Table 10:** Adversarial robustness performance metrics.

Attack type	AUC (Anomaly detection)	Trust score drop (%)	False acceptance reduction (%)
FGSM (ε = 0.1)	0.91	4.6	68.3
Replay Attack	0.87	6.2	72.5
Gaussian Noise (σ = 0.05)	0.89	3.2	61.4

### Ablation module-wise impact

Ablation experiments were conducted to disentangle the role of each architectural component—spatial encoder (CNN), temporal sequencer (LSTM), and latent uncertainty modeler (VAE). The trust-aware fusion exhibited optimal performance when all three were synergistically integrated. Notably, the LSTM module contributed significantly to contextual anomaly tracking, while VAE improved probabilistic trust calibration. The comparative results are outlined in Table 11, highlighting that partial configurations led to degraded F1 and AUC scores, affirming the necessity of multimodal fusion.

**Table 11:** Module-wise impact on anomaly detection and trust score accuracy.

Configuration	Anomaly F1-score (%)	Trust score AUC	SHAP value (Mean)
VAE Only	81.2	0.792	0.34
CNN + LSTM Only	85.4	0.803	0.47
CNN + LSTM + VAE (Full Model)	89.7	0.851	0.63

### Missing data imputation via VAE

In healthcare Internet of Things (IoT) environments, invalid sensor values and wireless transmission errors can result in missing or incomplete data, which can harm the reliability of models and diagnostic accuracy. To assess the advising usefulness of our proposed Variational Autoencoder (VAE)-based imputation mechanism, we compared it to two commonly used baseline methods: K-Nearest Neighbors (KNN) and Multiple Imputation by Chained Equations (MICE). We characterized the imputation performance using Rootejef eek mean square error (RMSE) on three physiological signals—ECG, PPG, and temperature—using missingness levels between 15 and 30%.

The results and performance metrics are summarized in Table 12. The VAE performed best with the lowest Autumn Solomon az Gna ae od isolated. Compared to KNN and MICE in terms of RMSE, with the VAE performing best across all signals. Given 20% missing ECG values, the RMSE for VAE was 0.048, which was lower than the KNN (0.067) and MICE (0.058) approaches. There were similar results for PPG and temperature data. Across the board, our proposed VAE method resulted in an averaged reduction of RMSE by 24.7% in comparison to KNN and by 17.3% in comparison to MICE. This highlights the strengths of VAE imputation in capturing latent signal patterns and reconstruct any signals consistently while maintaining signal characteristics. These findings affirm the VAE for use in real-time healthcare and with edge-device considerations, while providing high data fidelity.

**Table 12:** RMSE comparison for missing data imputation using VAE, KNN, and MICE.

Signal type	Missing rate (%)	VAE (Proposed)	KNN	MICE
ECG	20%	0.048	0.067	0.058
PPG	30%	0.063	0.081	0.074
Temperature	15%	0.027	0.036	0.032
Average	–	0.046	0.061	0.054

#### Logging and audit mechanisms

To ensure traceability and tamper resiliency when handling healthcare data, a logging mechanism is proposed that is cryptographically secure based on SHA-256 hash chains. Every system action occurs with a date and time on the action (e.g., detection of anomaly, decision to allow or reject access, and quarantine trigger) and is furthermore stored as a log with its own individual hash sequence to mitigate undetectable and illicit attempts to alter the data. The logs will have a periodic verification procedure using digital signatures and will be stored in a distributed ledger format for forensics certainly. Architecture will also allow for forward integrity and facilitate compliance directives in the healthcare domain so that audit trails are correct, verifiable, immutable, and resilient to tampering from both insiders and outsiders.

## Discussion

Comparative experimental verification of the suggested Secure and Trust-Aware Hybrid Deep Learning Framework for H-IoT produces some important findings about its performance, generalizability, and applicability. The combination of CNN for spatial encoding, LSTM for sequence modeling, and VAE for latent anomaly detection provides a robust, multimodal representation of the H-IoT data that detects anomalies with better performance (F1-score > 0.94) than conventional rule-based or single-model approaches. The application of Mahalanobis distance in the tamper detection inside the VAE latent space brings an additional resilience level by identifying distributional shifts even when there is adversary or subtle perturbation present. The innovation in this solution comes from the combination of multiple deep learning models with dynamically adaptive trust score based on contextual prediction confidence, past reliability, and anomaly activity. Unlike static trust models, our method adapts trust decisions in real-time, making it well-suited for volatile and resource-constrained environments such as healthcare IoT. The inclusion of differential privacy, AES encryption, and fog-based VAE offloading ensures data protection while preserving latency performance, an essential requirement for time-critical H-IoT applications. From a justification standpoint, the selected hybrid architecture and adaptive thresholds were empirically tuned across multi-signal datasets (ECG, PPG, temperature), ensuring cross-modal generalization. The performance remained consistent across both public (PhysioNet) and proprietary datasets, validating model transferability. Moreover, the trust score–based access control reduced false positives in permission decisions by over 30% compared to static ACL-based systems. The comparative evaluation also highlights superior detection accuracy and decision reliability across all tested scenarios. Traditional statistical models suffered from reduced sensitivity to temporal anomalies and poor generalization under noisy data, while our model sustained high precision (> 95%) even under adversarial tampering and signal degradation. Furthermore, the scalability experiments confirmed that inference latency remained within clinically acceptable thresholds (≤ 150 ms) even on edge devices, demonstrating deployment readiness. Finally, the error analysis uncovered edge cases such as high-frequency noise and overlapping anomalies where detection performance degraded marginally. This leaves room for potential inclusion of signal denoising autoencoders or attention in future.

## Conclusion and future scope

This paper presents a strong, flexible, and trusted Human-Centric Internet of Things (H-IoT) system by incorporating hybrid deep learning, anomaly detection, and trust-based access control schemes. The H-IoT architecture proposed using CNN for spatial representation, LSTM for temporal sequence modeling, and VAE for latent anomaly detection demonstrated high accuracy on various physiological signals. With the inclusion of context-adaptive trust score and tampering detection based on Mahalanobis distance, the system was further enhanced dynamically reacting to internal inconsistencies as well as attacks online. Multi-source dataset testing facilitated robust generalization, while latency and energy profiling facilitated scalability across devices. Among its substantial contributions is the novelty of integrating anomaly-aware latent modeling and multi-dimensional trust function towards the purpose of informing real-time access control decisions. This significantly enhances system reliability and resilience against traditional static or rule-based models of security with prospects towards real-world implementations in healthcare monitoring, wearable sensing, and real-time assistive technology. The quarantine model based on trust-aware locking down securely without entirely shutting down provides fine-grained accessibility span rather than binary choice. Although the outcome is affirmative, there are quite a few challenges to be addressed. For coordinated multi-signal attacks with high noise, the anomaly recall of the model reduces by a small percentage, a metric for the extent of increased robustness. Apart from this, with fog offloading static resource problems, coordination tuning of edges and fog is an open optimization problem. There will be work towards integrating light transformer architectures with signal attention, federated learning for privacy-enhanced multi-institution training, and adaptive reinforcement learning for dynamic trust score thresholding. Moreover, state-of-the-art model decision interpretability via explainable AI (XAI) modules will be essential to regulatory suitability and clinical adoption.

## Data Availability

The data required to reproduce the above findings are available within this research article.
